# A Review on *Sasa quelpaertensis*’s Phytochemical Profiles and Pharmacological Activities

**DOI:** 10.3390/plants15020319

**Published:** 2026-01-21

**Authors:** Varun Jaiswal, Hae-Jeung Lee

**Affiliations:** 1 Department of Food and Nutrition, College of BioNano Technology, Gachon University, 1342 Seongnam-daero, Sujeong-gu, Seongnam-si 13120, Republic of Korea; computationalvarun@gmail.com; 2 Institute for Aging and Clinical Nutrition Research, Gachon University, Seongnam-si 13120, Republic of Korea; 3 Department of Health Sciences and Technology, Gachon Advanced Institute for Health Sciences and Technology (GAIHST), Gachon University, Incheon 21999, Republic of Korea

**Keywords:** phytochemicals, herbal medicine, *Sasa quelpaertensis*, pharmacology, immune enhancement, anticancer, anti-inflammatory

## Abstract

*Sasa quelpaertensis*, a multipurpose bamboo plant endemic to Jeju Island in South Korea, is used by the population in traditional medicine for its anti-inflammatory, anti-diabetic, anti-gastritis, and diuretic activities. Studies have shown the potential of *S. quelpaertensis* against various diseases; its effects include anticancer, anti-obesity, anti-diabetic, anti-inflammatory, antibacterial, antiviral, antioxidant, antidepressant, immunomodulating, and hepatoprotective effects. Several bioactive phytochemicals, including *p*-coumaric acid, tricin, naringenin, and vanillic acid, have been identified in *S. quelpaertensis*, further emphasizing its pharmacological potential. Molecular studies have identified crucial pharmacological targets of *S. quelpaertensis*, such as adenosine monophosphate-activated protein kinase (AMPK) and nuclear factor kappa B (NF-κB) signaling. The major challenges are that most pharmacological activities have been observed only in the preclinical stage, and that a compilation of its phytochemicals and pharmacological activities is missing from the literature. The studies with incomplete extract characterization or standardization limit the comparability across studies. Identification of active phytochemicals for specific activities and large-scale clinical trials for the majority of pharmacological effects are suggested. This review not only compiles the phytochemicals and pharmacological properties of *S. quelpaertensis* but also highlights current gaps and proposes solutions for its development as a therapeutic agent and/or supplement against major diseases.

## 1. Introduction

*Sasa quelpaertensis* Nakai, also known as Jeju-Joritdae, is a bamboo plant endemic to Jeju Island, South Korea, where it is primarily grown in the Halla Mountain region. *S. quelpaertensis* belongs to the family Poaceae; the botanist Takenoshin Nakai named the plant based on specimens collected during a survey of vegetation inhabiting Halla Mountain. The abundance of *S. quelpaertensis* in the Halla Mountain region is high, covering approximately 76% of the northern slopes of the Mount Halla National Park and posing a threat to the ecosystem diversity in the region [1]. Thus, efforts are required to develop *S. quelpaertensis* for various applications, including beverages (herbal tea), ornamental gardening, traditional medicine, and recently as a roughage source for livestock [1]. The traditional bamboo tea made from the leaves of *S. quelpaertensis* has long been used for medicinal purposes by the local population. These folk medicinal applications are based on the anti-inflammatory, anti-diabetic, anti-gastritis, and diuretic effects of the plant [2,3]. However, the pharmacological properties of *S. quelpaertensis* have been scientifically studied over the past few decades.

The main potential medicinal properties of *S. quelpaertensis* include anticancer [4], anti-obesity [5], anti-diabetes, anti-inflammatory, antimicrobial [6], antioxidant [7], antidepressant [8], immunomodulatory, and hepatoprotective effects [9].

Among these pharmacological properties, anti-inflammatory, antioxidant, and immunomodulatory effects can contribute to other important pharmacological activities, including anticancer activity, which indicates the strong potential of developing this endemic plant as a supplement and/or therapeutic candidate [10,11,12].

In several studies, the effect of *S. quelpaertensis* was better than that of the primary phytochemicals reported in *S. quelpaertensis*, which suggests the need for further research into its minor components that may be important for its activity [13]. Various pharmacological activities and numerous phytochemicals have been identified in *S. quelpaertensis*.

However, a comprehensive compilation of the pharmacological properties and phytochemicals of *S. quelpaertensis* is missing in the literature. The current review not only compiles the phytochemicals and pharmacological properties of *S. quelpaertensis* but also highlights the current gaps and proposed solutions for the development of *S. quelpaertensis* as a therapeutic and supplement against critical diseases.

## 2. Electronic Literature Search Strategy

A comprehensive compilation of the phytochemicals and pharmacological activities of *S. quelpaertensis* is unavailable in the literature. Therefore, we searched for associated studies in major electronic databases (PubMed, Scopus, ResearchGate, and Web of Science). Significant keywords associated with *S. quelpaertensis* and its pharmacological properties, such as *S. quelpaertensis*, Jeju Joritdae, diabetes, obesity, cancer, pharmacology, phytochemicals, anti-inflammation, and depression, were searched using relevant combinations on ResearchGate, PubMed, Scopus, and Web of Science. Related documents in English from the search hits, including research articles, review articles, books, and book chapters, were considered in this study. Initially, the documents were screened by reviewing their abstracts or summaries. The documents reporting phytochemical and/or pharmacological properties of *S. quelpaertensis* were included in the study; all other documents were excluded. A total of 41 articles from *S. quelpaertensis* were included in the study. However, to maintain the flow of the review, the current work includes literature describing the plant, properties of phytochemicals, the importance of related diseases, and other associated information.

## 3. Botanical Description

*S. quelpaertensis*, a dwarf bamboo grass, belongs to the grass family (Poaceae) and the bamboo genus *Sasa*. *S. quelpaertensis* is a perennial rhizomatous plant that can reach heights up to 1 m [14]. *S. quelpaertensis* begins growing in April, with peak growth and yield in August [1].

Its culms (stems) are solid, thick, and erect, with an average diameter of approximately 0.6 inches. The leaves are long and narrow with pointed tips (oblong-lanceolate) and are dark green with a distinct cream-colored margin that is often pale.

The leaf is approximately 2.8 to 7.9 inches long, and its width ranges from 1 to 2 inches. The part of the leaf that wraps around the culm (leaf sheaths) is densely covered in stiff hairs, imparting a rough texture [14], and the edges of the leaf begin to fade near September [1].

The inflorescence of the plant is a panicle, which is a branched structure bearing spikelets. The spikelets arise from the leaf axils (the angle between the leaf and stem), and they are primarily covered with brownish-red bracts (modified leaves). *S. quelpaertensis* is widely distributed across the slopes of Mount Halla, often forming dense understory layers, particularly in the mid-mountainous and subalpine zones [1].

## 4. Phytochemicals of *S. quelpaertensis*

Primarily, the phytochemicals were identified in *S*. *quelpaertensis* to decipher their role in its pharmacological activity. A diverse set of pharmacologically active phytochemicals was identified in the different parts of the plants. In an initial study, bioactivity-guided isolation revealed the presence of five compounds (3-*O*-*p*-coumaroyl-1-(4-hydroxy-3,5-dimethoxyphenyl)-1-propanone, 3-*O*-*p*-coumaroyl-1-(4-hydroxy-3,5-dimethoxyphenyl)-1-*O*-β-gulcopyranosylpropanol, *p*-coumaric acid, *N*-*p*-coumaroylserotonin, and *N*-feruloylserotonin) with tyrosinase inhibition activities in the ethyl acetate fraction of the leaf ethanol extract of *S. quelpaertensis* (SQLEEEA; Table 1). The compounds were isolated using vacuum liquid chromatography and the Sephadex LH-20 method. The compounds were characterized by ^1^H and ^13^C NMR spectroscopy. Further purification and isolation revealed five compounds with tyrosinase inhibitory activity. Among the isolated compounds, serotonin derivatives show the highest tyrosinase inhibitory activity [15].

Additionally, the researcher identified a new compound ((*E*)-3-hexenyl-β-glucopyranoside) and nine previously known compounds (4-hydroxybenzoic acid, 3-hydroxy-1-(4-hydroxy, 3,5-dimethoxyphenyl)-1-propanone, saikochromone A, tricin-7-*O*-glycoside, tricitin-3′,4′,5′-tri-*O*-methyl-7-*O*-β-glucopyranoside, isoorientin, daucosterol, lutein, and tricin) from the ethyl acetate fraction of *S. quelpaertensis* leaf methanol extract (SQLEMEA) [3].

Phenolic compounds are believed to be responsible for the pharmacological activities of plants. Thus, the total phenol content of *S. quelpaertensis* leaf extract in different ethanol concentrations and distilled water was analyzed to determine the optimal phenolic content and biological activity [16]. The phenolic content was high (990,010 ± 28,900 µg Gallic acid equivalent (GAE)/g) in 40% ethanol concentration and low (653.80 ± 13.14 GAE/g) in 100% ethanolic solvent.

Subsequently, the total phenolic and flavonoid contents of the leaf extracts in *n*-hexane, chloroform, *n*-butanol, and ethyl acetate fractions were examined. The highest phenol (95,000 ± 1000.0 µg GAE/g) and flavonoid (262,400 ± 4100 µg naringin equivalent (NGE)/g) contents were found in the ethyl acetate and *n*-butanol fractions, respectively [17]. Furthermore, thirteen compounds were isolated from these extracts, of which the following seven were isolated for the first time from *S. quelpaertensis* (Table 1): salicylic acid, syringaldehyde, methyl cis-*p*-hydroxycinnamate, methyl trans-*p*-hydroxycinnamate, 2,3-dihydroxypropyl 9Z,12Z-octadecadienoate, (+)-(6S,7aS)-epilolide, and (−)-(6R,7aS)-loliolide. These compounds were identified using ^1^H and ^13^C NMR methods [17].

**Table 1:** Phytochemicals reported in *S. quelpaertensis*.

Sr. No.	Compound Name	Amount (µg/g)	Type of Extract/Fraction	Chemical Class	Part of a Plant	References
1	(*E*)-3-Hexenyl- β-glucopyranoside	NP	Ethyl acetate fraction of the extract	Glycoside	Leaf	[3]
2	4-Hydroxybenzoic acid	10.92	Ethyl acetate fraction of the extract	Hydroxybenzoic acid	Leaf	[3]
3	3-Hydroxy-1-(4-hydroxy 3,5-dimethoxyphenyl)-1-propanone	13.07	Ethyl acetate fraction of the extract	Phenylpropanoid	Leaf	[3]
4	Saikochromone A	9.84	Ethyl acetate fraction of the extract	Chromone derivative	Leaf	[3]
5	Tricin-7-O-glycoside	18.61	Ethyl acetate fraction of the extract	Flavonoid	Leaf	[3]
6	Tricitin-3′,4′,5′-tri-Omethyl- 7-O-β-glucopyranoside	12.92	Ethyl acetate fraction of the extract	Flavonoid	Leaf	[3]
7	Isoorientin	49.84	Ethyl acetate fraction of the extract	Flavonoid	Leaf	[3]
8	Daucosterol	20.76	Ethyl acetate fraction of the extract	Sterol Glycoside	Leaf	[3]
9	Lutein	76.92	Ethyl acetate fraction of the extract	Carotenoid	Leaf	[3]
10	Salicylic acid	NP	70% ethanol extract fraction	Hydroxybenzoic acid	Leaf	[17]
11	Syringaldehyde	NP	70% ethanol extract fraction	Hydroxybenzoic acid	Leaf	[17]
12	Methyl cis-p-hydroxycinnamate	NP	70% ethanol extract fraction	Hydroxycinnamic acid	Leaf	[17]
13	Methyl trans-p-hydroxycinnamate	NP	70% ethanol extract fraction	Hydroxycinnamic acid	Leaf	[17]
14	*p*-Coumaric acid	5090	Hot water extract fraction	Hydroxycinnamic acid	Leaf	[18]
		6450	70% ethanol extract fraction		culm	
15	2,3-Dihydroxypropyl 9Z, 12Z-octadecadienoate	NP	70% ethanol extract fraction	Glycerolipid	Leaf	[17]
16	(+)-(6S, 7aS)-Epilolide	NP	70% ethanol extract fraction	Monoterpenoid	Leaf	[17]
17	(−)-(6 R, 7aS)-Loliolide	NP	70% ethanol extract fraction	Monoterpenoid	Leaf	[17]
18	Naringenin	NP	70% ethanol extract fraction	Flavonoid	Leaf	[17]
19	Tricin	1970	70% ethanol extract fraction	Flavonoid	Leaf	[18]
		560	70% ethanol extract fraction		culm	
20	Tricin 7-O-b-D-glucopyranoside	NP	70% ethanol extract fraction	Flavonoid	Leaf	[17]
21	Vanillic acid	2100	Water extract	Hydroxybenzoic acid	Leaf	[19]
22	Isoorientin	1560	Water extract	Flavonoid	Leaf	[19]
23	3-*O*-*p*-coumaroyl-1-(4-hydroxy-3,5-dimethoxyphenyl)-1-propanone	12.97	Ethyl acetate fraction of the methanol extract	Coumaroyl metabolite	Leaf	[15]
24	3-*O*-*p*-coumaroyl-1-(4-hydroxy-3,5-dimethoxyphenyl)-1-*O*-β-gulcopyranosylpropanol	82.91	Ethyl acetate fraction of the methanol extract	Coumaroyl metabolite	Leaf	[15]
25	N-feruloylserotonin	28.18	Ethyl acetate fraction of the methanol extract	Alkaloid	Leaf	[15]
26	N-*p*-coumaroylserotonin	7.6	Ethyl acetate fraction of the methanol extract	Alkaloid	Leaf	[15]
27	Rutin	24.84	Ethyl acetate fraction of the water extract	Flavonoid	Leaf	[20]
28	Taxifolin	27.91	Ethyl acetate fraction of the water extract	Flavonoid	Leaf	[20]
29	Naringin	1.34	n-hexane fraction of the water extract	Flavonoid	Leaf	[20]
30	Myricetin	59.96	Ethyl acetate fraction of the water extract	Flavonoid	Leaf	[20]
31	Quercetin	11.63	Chloroform fraction of the water extract	Flavonoid	Leaf	[20]
32	Luteolin	2.77	Ethyl acetate fraction of the water extract	Flavonoid	Leaf	[20]
33	Apigenin	2.93	Chloroform fraction of the water extract	Flavonoid	Leaf	[20]
34	Kaempferol	6.56	Ethyl acetate fraction of the water extract	Flavonoid	Leaf	[20]
35	Rhamnetin	19.58	Ethyl acetate fraction of the water extract	Flavonoid	Leaf	[20]
36	Nobiletin	5.43	Ethyl acetate fraction of the water extract	Flavonoid	Leaf	[20]
37	Tangeretin	1.24	Ethyl acetate fraction of the water extract	Flavonoid	Leaf	[20]

NP: not provided.

Seasonal variations in plant phytochemicals can affect product quality and the application of the plants. Knowledge of seasonal variations in phytochemicals is important for determining the best harvesting time for product standardization in different industries. Thus, to determine the optimal harvesting time, studies evaluated important phytochemicals and total phenolic and flavonoid contents in the leaf and culm extracts of *S. quelpaertensis* in different seasons.

In one study, ethanol and water extracts of leaves and culms were analyzed for different seasons [18]. The same procedure was followed in the preparation of the extract from the samples of all seasons. The leaves were crushed and sieved using a wire mesh sieve (aperture size, 1.40 mm) and refluxed with distilled water three times at room temperature for a period of 24 h. High-performance liquid chromatography (HPLC) was used to analyze phenolic acids and flavonoids in the extracts. *p*-Coumaric acid was more abundant in the culms than in the leaves, and the highest (in September, 6450 µg/g) was in the water extract of the culms. Conversely, tricin was present in higher amounts in ethanol extracts than in water extracts and was more abundant in the leaf than in the culm [18]. In a later study, the water extract of the leaves showed the highest content of vanillic acid and isoorientin in May compared with the other extracts [19].

The TP level was higher in the leaf than in the culm, and the highest (50,200 µg GA/g in December) was in the ethanol extract. Similarly, TF levels were higher in the leaves than in the culm, and the highest (72.600 µg NG/g in December) was in the ethanol extract [18]. Mostly, the optimization of *S. quelpaertensis* extraction relies mainly on two principal pharmacologically active constituents: tricin and *p*-coumaric acid. It is crucial to note, however, that the different pharmacological activities are not solely dependent on these two. A diverse range of other identified phytochemicals, such as saikochromone A, isoorientin, daucosterol, lutein, naringenin, vanillic acid, methyl cis/trans-p-hydroxycinnamate, serotonin derivatives (e.g., N-feruloylserotonin, and N-*p*-coumaroylserotonin), can also play a significant role in the plant’s therapeutic potential. The pharmacological properties of *S. quelpaertensis* are explained in the next section.

## 5. Pharmacological Effects of *S. quelpaertensis*

The medicinal properties of *S. quelpaertensis* are known from the traditional medicinal practice of using its leaves as an anti-inflammatory, anti-diabetic, anti-gastritis, and diuretic agent [2,3] and as an herbal tea by the local people of Jeju Island, South Korea. Scientific evaluation of *S. quelpaertensis* leaves and culms (in a few cases) revealed its therapeutic potential against multiple diseases, including infectious (bacterial, viral, and fungal) and non-infectious diseases, owing to its anticancer [4], anti-obesity [5], anti-diabetic, anti-inflammatory, antioxidant [7], antidepressant [8], immunomodulatory, and hepatoprotective effects [9].

### 5.1. Antimicrobial Activity S. quelpaertensis Extracts

Antimicrobial drug resistance poses a significant challenge to the treatment of infectious diseases. Plant-based antimicrobials have emerged as a potential solution to drug resistance, highlighting the need to discover new plant products with antimicrobial properties [21]. The antimicrobial efficacy of *S. quelpaertensis* extracts was evaluated in vitro against a range of viral, bacterial, and fungal pathogens.

One study estimated the total phenolic content of *S. quelpaertensis* leaf ethanol extract (SQLEE) and the minimum inhibitory concentration of the extract against three Gram-positive bacteria (*Bacillus cereus*, *Staphylococcus epidermidis*, and *Staphylococcus aureus*), three Gram-negative bacteria (*Pseudomonas aeruginosa*, *Escherichia coli*, and *Proteus vulgaris*), and two yeasts (*Pichia jadnii* and *Candida albicans* (KCTC 7965)). The 80% ethanol extract was effective against most of the bacterial species used in the study (Table 2) and *Pichia jadnii*; however, none of the extracts were effective against *Candida albicans* [16]. The findings suggest that SQLEE has higher potential against bacterial pathogens than against fungal.

The antiviral activity of *S. quelpaertensis* leaf extract (SQLE) was studied against porcine reproductive and respiratory syndrome virus (PRRSV), which is associated with respiratory illness in young piglets and pregnancy failure in pregnant sows [6]. PRRSV grows favorably in porcine alveolar macrophages (PAMs). Hence, an immortalized PAM cell line (PAM-KNU) was used to study the effects of SQLE on PRRSV. Pretreatment with SQLE inhibited PRRSV through cytopathic effects and N-protein-specific monoclonal antibody (MAb) and achieved 80% inhibition of (3 mg/mL) PRRSV infection in PAM-KNU cells. Post-infection introduction of SQLE at different time points in PAM-KNU mice revealed that SQLE acts during the initial phase of infection. Furthermore, the effect of SQLE on viral replication was studied using viral titers, which showed dose-dependent inhibition of the release of viral progeny. The effect of SQLE on viral entry into PAM-KNU was analyzed using an internalization assay, which suggested that SQLE does not inhibit the entry of the virus. Western blot (WB) analysis revealed significant (90%) suppression of the PRRSV N protein following treatment with SQLE. Similarly, RT-PCR analysis showed that 5 mg/mL SQLE suppressed the genomic (90%) and subgenomic (80%) mRNA expression of PRRSV. Northern blot analysis confirmed the suppression of PRRSV transcription, which was more than 50% at a dose of 1 mg/mL and almost completely inhibited at 5 mg/mL. However, 5 mg/mL is the high dose that may cause practical limitations in in vivo and clinical studies. Additionally, the anti-inflammatory properties of SQLE were also analyzed in this study. SQLE treatment suppressed the expressions of IL-1α, IL-6, IL-8, IL-15, TNF-α, and AMCF-1, which were increased owing to PRRSV infection. Conversely, SQLE treatment enhanced the expression of chemokine genes such as MCP-1 and RANTES, which had been suppressed by PRRSV infection. SQLE treatment increased the expression of antiviral genes, including Mx1 and ISG-15, interferon regulatory factors (IRFs), and toll-like receptors (TLRs). This study suggested that the antiviral activity of SQLE may also be supported by its anti-inflammatory activity [6].

### 5.2. Tyrosinase Inhibitory and Anti-Melanogenesis Activity

Tyrosinase is the key enzyme in melanin production, which is associated with the pigmentation responsible for darker skin color; thus, inhibition of tyrosinase can produce a skin-whitening effect [36]. The tyrosinase inhibitory activity of *S. quelpaertensis* extracts and isolated compounds was evaluated in vitro experiments.

#### 5.2.1. In Vitro Tyrosinase Inhibitory Activity of Extracts

**Figure 1:**
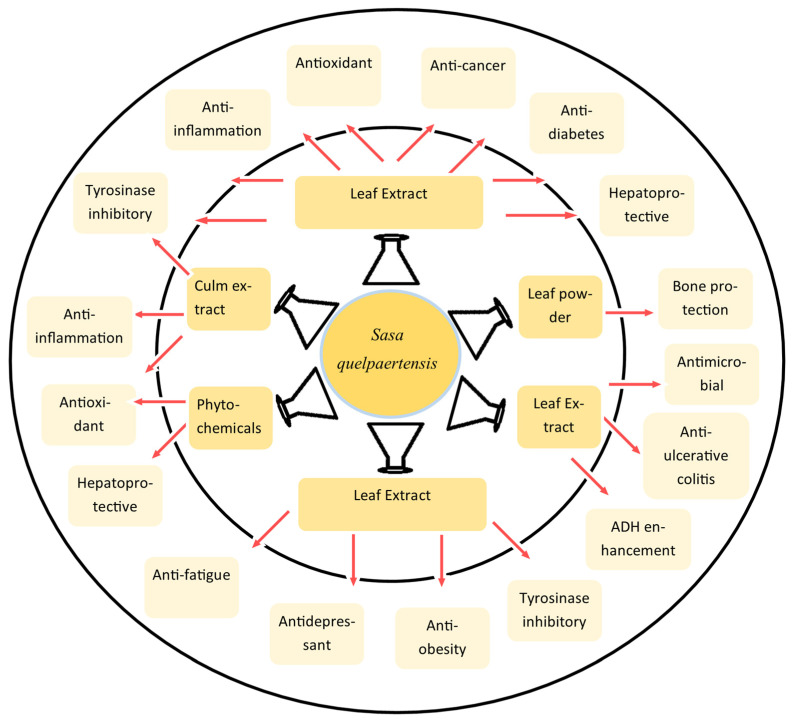
The pharmacological effects of *S. quelpaertensis*.

In an initial study, the leaf and culm extracts were prepared in 70% ethanol to analyze their anti-melanogenesis activity on α-melanocyte-stimulating hormone (α-MSH)-stimulated murine melanoma B16⁄F10 cells [37] (Figure 1). Treatment with *S. quelpaertensis* culm ethanol extract (SQCEE) and SQLEE significantly suppressed melanin synthesis in α-MSH-stimulated murine melanoma B16⁄F10 cells; SQCEE showed a greater suppressive effect than SQLEE. Fractions of the SQCEE in methylene chloride, ethyl acetate, butanol, and water were studied for their anti-melanogenesis activity in murine melanoma B16⁄F10 cells; the ethyl acetate and butanol fractions inhibited melanogenesis better than SQCEE. Furthermore, the ethyl acetate and butanol fractions suppressed the tyrosinase activity, which had been increased because of the α-MSH stimulation.

Furthermore, tyrosinase inhibitory activity was determined for different fractions of methanol-based SQLE in ethyl acetate, *n*-butanol, and *n*-hexane. A significant, dose-dependent tyrosinase inhibitory activity was observed for the ethyl acetate fraction. This ethyl acetate fraction was subsequently utilized to identify its bioactive compounds.

Later, after estimating the total phenolic content (990,010 ± 28,900 µg GAE)/g) of the leaf extract, its tyrosinase inhibitory activity was studied using the l-3,4-dihydroxyphenylalanine (l-DOPA) quinone-based method, with arbutin as a comparative control [16]. Dose-dependent inhibition of tyrosinase activity was observed for all extracts (0–100% ethanol extracts), with the 80% ethanol extract exhibiting the highest activity (Table 2). Recently, different extraction conditions were optimized using the response surface methodology to obtain the leaf extract with the maximum tyrosinase inhibitory activity [7]. A maximum of 51.00 ± 1.80% tyrosinase inhibitory activity was achieved with the selected optimal condition. Following the further development of SQLE as a cosmetic ingredient, the optimized extraction conditions for effective tyrosinase inhibitory and anti-melanogenesis activities can be applied to in vivo animal studies.

#### 5.2.2. In Vitro Tyrosinase Inhibitory Activity of Isolated Compounds

The methanolic extract of *S. quelpaertensis*, which previously demonstrated dose-dependent tyrosinase inhibition, was selected for further analysis to identify its bioactive constituents. Further purification and isolation revealed five compounds that inhibited tyrosinase. These compounds, which include 3-*O*-*p*-coumaroyl-1-(4-hydroxy-3,5-dimethoxyphenyl)-1-propanone (IC_50_ = 0.055 mM), 3-*O*-*p*-coumaroyl-1-(4-hydroxy-3,5-dimethoxyphenyl)-1-*O*-*β*-gulcopyranosylpropanol (IC_50_ = 0.053 mM), N-feruloylserotonin (IC_50_ = 0.026 mM), N-*p*-coumaroylserotonin (IC_50_ = 0.027 mM), and *p*-coumaric acid (IC_50_ = 0.12 mM), were present in the ethyl acetate fraction at concentrations of 12.97, 82.91, 28.18, and 7.6 µg/g, respectively. Among the isolated compounds, the serotonin derivatives *N*-*p*-coumaroylserotonin and *N*-feruloylserotonin showed high tyrosinase inhibitory activity [15]. Thus, the relatively high concentrations and potent tyrosinase inhibitory activities of *p*-coumaroylserotonin and *N*-feruloylserotonin compared to other studied compounds support their roles as major contributors to the extract’s tyrosinase inhibitory activity.

### 5.3. Estimation of Alcohol Dehydrogenase (ADH)- and Aldehyde Dehydrogenase (ALDH)-Inducing Activity of S. quelpaertensis Extracts

ADH- and ALDH-inducing activity can relieve alcohol-associated hangovers and prevent other alcohol-associated diseases such as cancer. The recent success of herbal medicines in inducing ADH and ALDH has inspired the development of plant-based interventions against hangover [38]. In one study, after estimating the total phenolic content of SQLEE, the ADH-inducing activities of the extract and its fractions in ethyl acetate, chloroform, *n*-butanol, *n*-hexane, and water were evaluated [26]. Similar to the tyrosinase inhibitory activity, the ADH-inducing activity was dose-dependent and was the highest in the 80% ethanol extract (Table 2).

Similarly, the 80% ethanol extract of *S. quelpaertensis* leaves and its fractions were evaluated for ALDH-inducing activity. ALDH activity was dose-dependently enhanced by the SQLEE treatment and was the highest in the SQLEEEA [26]. The effective inducing activity for both ADH and ALDH strengthens the potential of SQLEE against alcohol-associated hangovers and diseases, which may be explored in further research.

### 5.4. Antioxidant Activity of S. quelpaertensis Extracts

Oxidants such as free radicals can contribute to various health problems and aging. Antioxidants help maintain overall health by fighting various diseases [39,40]. Plant products are considered good sources of antioxidants that can be used as therapeutics or supplements.

In an initial study, the leaf extract was prepared in methanol to analyze its antioxidant activity through the DPPH method along with its fraction in hexane, butanol, water, and ethyl acetate. The maximum and minimum antioxidant activity was observed in the butanol fraction and the methanol extract, respectively [27]. The minimum DPPH activity (IC_50_ = 862.5 ± 6.4 μg/mL) was observed in the methanol extract, which was insubstantial compared to the butanol fraction (Table 2).

In one study, the antioxidant activity of the leaf extract of *S. quelpaertensis* was studied using DPPH, nitric-oxide-scavenging, ferrous-ion-chelation, and reducing-power assays [23]. Water and ethanol extracts were prepared to evaluate the antioxidant activity. For all methods (DPPH, hydrogen-peroxide inhibition, nitric-oxide-scavenging, ferrous-ion-chelation, and reducing-power assays), the water and ethanol extracts showed effective antioxidant activities that were higher in the ethanol extract, which corroborated the presence of higher TP and TF contents in the ethanol extract than in the water extract [23].

To determine the best harvesting time for *S. quelpaertensis* based on biological activity, the antioxidant activity of leaves and culms harvested during different seasons was studied using the DPPH and ABTS methods. The antioxidant activities determined using the DPPH method were higher in the leaf extract than in the culm extract. The antioxidant activities of the leaf extracts were similar in both water and ethanol extracts and were maximum in November and December (Table 2). However, minimum DPPH activity (IC_50_ = 785.5 μg/mL) was observed in September, which was insubstantial compared to November and December (Table 2). Similarly, the antioxidant activity, determined using the ABTS method, was higher in the leaf extract than in the culm extract. The ethanol extract exhibited the maximum activity (IC_50_ = 72.6 ± 3.6 μg/mL) in December (Table 2). Subsequently, a reduction in reactive oxygen species (ROS) generation was studied in H_2_O_2_-stimulated PC12 neuronal cells [19]. The application of the leaf water extract inhibited the production of ROS in the cells, and the leaves harvested in May were the most effective (Table 2). The reduction in ROS was correlated with the isoorientin concentration in the extract, indicating that isoorientin plays a role in the suppression of intracellular ROS. It can be concluded that the highest antioxidant activity (suppression of intracellular ROS) was observed in May. This corresponds to the highest TPC (298,000 µg GAE/g), TFC (240,000 µg QE/g), isoorientin (1580 µg/g), and vanillic acid (2100 µg/g) contents in the SQLE.

Recently, different extraction conditions were optimized using response surface methodology to obtain the leaf extract with the maximum antioxidant activity [7]. A maximum of 83.65 ± 1.56% antioxidant activity, as determined using the DPPH method, was achieved under the selected optimal conditions. The leaf extract exhibited better antioxidant activity than the culm extract, and response surface methodology successfully optimized the extraction conditions. These selected conditions are essential for further studies of the enzymatic antioxidant activity of SQLE to strengthen its pharmacological potential.

### 5.5. Anti-Inflammatory Activity

**Figure 2:**
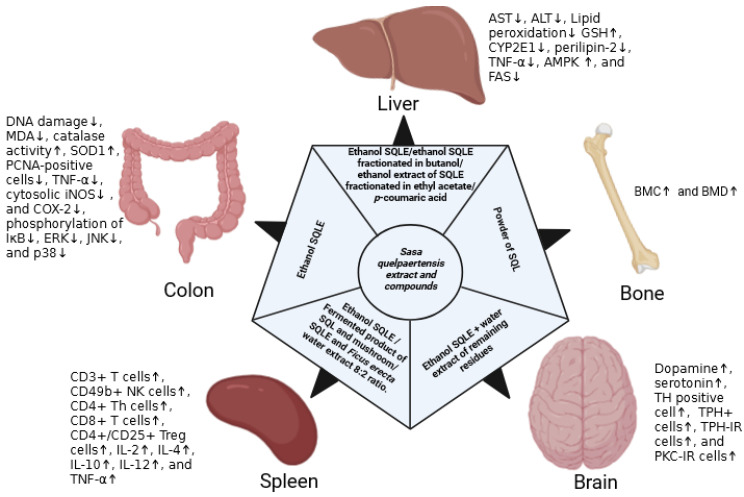
Protective effect of *S. quelpaertensis* on different organs (ALT: alanine aminotransferase; AST: aspartate aminotransferase; BMC: bone mineral concentration; BMD: bone mineral density; MDA: malondialdehyde; PCNA: proliferating cell nuclear antigen; iNOS: inducible isoform of nitric oxide synthase enzyme; SQL: *S. quelpaertensis* leaf; SQLE: *S. quelpaertensis* leaf extract; ↑: up-regulation; ↓: down-regulation).

Inflammation is associated with several diseases and adverse health conditions; therefore, anti-inflammatory agents may exert various health-promoting effects (Figure 2) [41,42]. Inspired by the traditional use of *S. quelpaertensis* as an anti-inflammatory therapeutic, various studies have investigated the anti-inflammatory activities of the isolated compounds, leaves, and culm extracts.

#### 5.5.1. Anti-Inflammatory Activity of Extracts

The anti-inflammatory activity of *S. quelpaertensis* leaf extract was studied using an in vitro inflammatory system, i.e., inflammation induced in RAW 264.7 cells using lipopolysaccharide (LPS) [28]. The hot water extract of the leaves was found to inhibit the NO production in the LPS-induced RAW 264.7 cells in a dose-dependent manner. Furthermore, the extract was found to inhibit the inducible isoform of nitric oxide synthase enzyme (iNOS) in a dose-dependent manner, which suggests that the suppression of NO production by the extract may be attributed to the inhibitory activity of the iNOS enzyme. Additionally, the hot water extract significantly decreased the protein expression of iNOS, which had been increased by LPS administration. Luciferase-based analysis showed that LPS increased (>12.5 fold) the activation of nuclear factor kappa B (NF-κB), which is crucial in inflammation. The hot water extract significantly suppressed the activation of NF-κB by LPS in the RAW 264.7 cells [28]. Finally, the hot water extract protected against LPS-induced cell death in the RAW 264.7 cells, as observed by measuring lactate dehydrogenase (LDH), a marker for cell membrane integrity.

Critical inflammatory diseases such as inflammatory bowel disease, which includes ulcerative colitis (UC) and Crohn’s disease, are mainly characterized by chronic inflammation of the gastrointestinal tract. One study explored the anti-inflammatory activity of *S. quelpaertensis* against these diseases by analyzing its effects on inflammation in the gastrointestinal tract [30]. For this purpose, an in vitro co-culture system, comprising intestinal epithelial Caco-2 cells and RAW 264.7 macrophages, was established. The 70% ethanol leaf extract at different concentrations suppressed cytokine expression in a co-culture medium consisting of intestinal epithelial Caco-2 cells and RAW 264.7 macrophages. Pretreatment with 70% ethanol leaf extract significantly suppressed NO production in the co-culture model, which had been increased by LPS. Similarly, the LPS-induced increase in the expression of cytokines (PGE_2_, IL-1β, and IL-6) was significantly suppressed by the pretreatment with 70% ethanol leaf extract. Similarly, pretreatment with the extractsuppressed the protein expression of iNOS and COX-2, which had been enhanced by LPS treatment [30]. The hyperphosphorylation of IκBα is associated with NF-κB activation through LPS. WB analysis showed that the extract suppressed the LPS-induced hyperphosphorylation of IκBα in a dose-dependent manner. Additionally, treatment with the extract significantly suppressed the expression of TNF-α mRNA, which had been increased by LPS.

The anti-inflammatory activity of *S. quelpaertensis* has been studied to determine the optimal harvesting time for *S. quelpaertensis* by evaluating its inhibitory effect on nitrite accumulation in LPS-stimulated RAW 264.7 cells [18]. The maximum anti-inflammatory activity was observed in the ethanol extract of leaves harvested in October (Table 2). Additionally, ethanol extracts exhibited a higher antioxidant activity than water extracts from both leaves and culms.

#### 5.5.2. Anti-Inflammatory Activity of Isolated Compounds

The previously isolated compound (3-*O*-*p*-Coumaroyl-1-(4-hydroxy-3,5-dimethoxyphenyl)-1-*O*-β-D-gulcopyranosylpropanol (3CPG) from the leaves of *S. quelpaertensis* was studied for its anti-inflammatory activity against LPS-induced RAW 264.7 cells [29]. 3CPG suppressed NO production in a dose-dependent manner in the LPS-induced RAW 264.7 cells. Furthermore, it showed a dose-dependent inhibition of iNOS gene expression, which suggested NO inhibition as its mechanism [29]. Additionally, the compound showed a dose-dependent suppression of the expression of inflammatory prostaglandin E_2_ (PGE_2_) molecules, such as PGE_2_, which had been increased by LPS treatment. PGE_2_ is an inflammatory mediator produced by COX-2 activity; thus, COX-2 expression was evaluated as well. 3CPG inhibited COX-2 in a dose-dependent manner, suggesting that PGE_2_ suppression by 3CPG might be attributed to the inhibition of COX-2 expression, which had been enhanced by LPS induction. Among the proinflammatory cytokines, IL-6 production, which had been enhanced by LPS induction, was suppressed by 3CPG in a dose-dependent manner.

In another study, the anticancer activities of SQLEE and its two major constituents, tricin and *p*-coumaric acid, suppressed cytokine expression in a co-culture medium consisting of intestinal epithelial Caco-2 cells and RAW 264.7 macrophages. Pretreatment with an equivalent amount of tricin (1 μM) and *p*-coumaric acid (2.4 μM) present in 400 μg/mL extract inhibited the LPS-induced NO production and IL-6 expression. *p*-Coumaric acid significantly suppressed the expression of IL-1β. Similarly, individual pretreatment with the extract, tricin, and *p*-coumaric acid suppressed the protein expression of iNOS and COX-2, which had been enhanced by LPS treatment [30]. Similarly, *p*-coumaric acid suppressed the LPS-induced phosphorylation of IκBα, but to a lesser extent compared with the extract [30]. Additionally, individual treatment with *p*-coumaric acid also significantly suppressed the expression of TNF-α mRNA, which had been increased by LPS. In the anti-inflammatory studies, the activity of the extract was superior to that of its individual studied constituents (tricin and *p*-coumaric acid) across nearly all parameters. Since the compounds were tested at concentrations equivalent to those found in the extract, it can be concluded that the extract exerts a synergistic effect.

### 5.6. Anti-UC Activity of S. quelpaertensis Extracts

Consistent with their anti-inflammatory activities, various plant-based products have shown therapeutic potential against UC, an inflammatory disease of the intestine [43].

**Figure 3:**
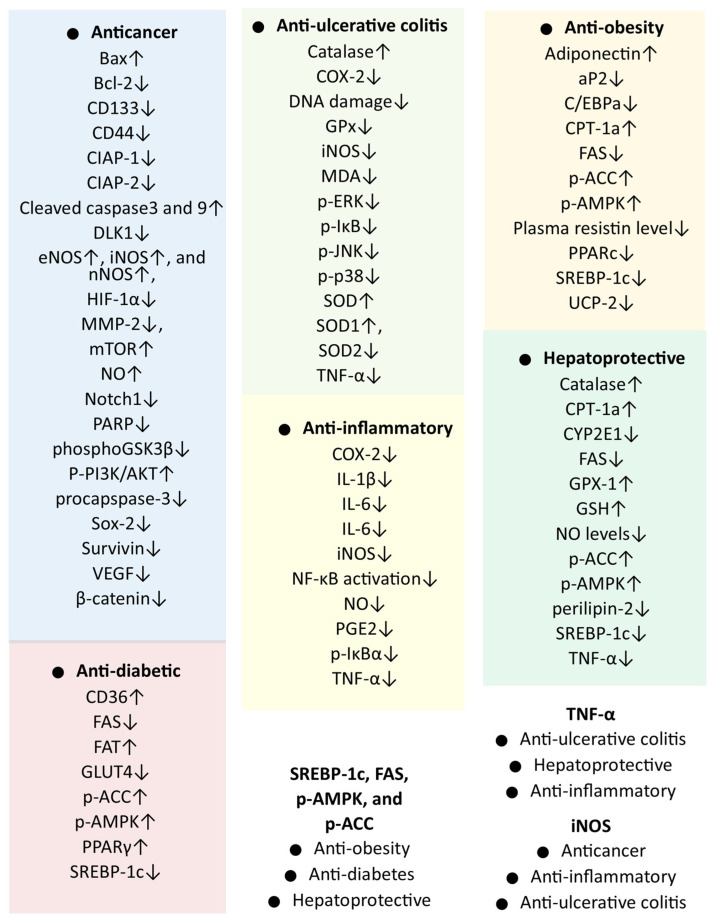
Different targets/markers were found to be associated with the pharmacological activities of *Sasa quelpaertensis*. (Studied genes common in more than two activities are listed with a white background, and upward (↑) and downward (↓) arrows represent significantly up-regulated and down-regulated genes, respectively).

On the basis of the known anti-inflammatory activity of SQLEE, its potential against UC was studied. Two doses (100 and 300 mg/kg) of SQLEE were evaluated in a mouse model of dextran sulfate sodium (DS)-induced colitis [44]. In this study, the incidence of diarrhea, bloody stool, and weight loss increased in the DS group, which increased the disease activity index (DAI). In contrast, the DAI score was significantly reduced in the treatment groups in a dose-dependent manner. SQLEE treatment improved the shrinkage of colon length due to DS administration; however, this effect was only significant in the 300 mg/kg dose group. A histological study of the colon tissue revealed that DS causes mucosal atrophy, submucosal thickening, alterations in membrane architecture, and degeneration of the intestinal crypts [44]. SQLEE suppressed tissue damage and promoted crypt regeneration. To study intestinal inflammation and enterocyte proliferation, proliferating cell nuclear antigen (PCNA)-positive cells were studied. The DS group showed a high PCNA index labeling, which was reduced by the SQLEE treatment. The serum expression of the inflammation mediator TNF-α was increased in the DS group but was reduced following SQLEE treatment, according to ELISA and RT-PCR studies. Similarly, the levels of cytosolic iNOS and COX-2, which were enhanced in the DS treatment group, were suppressed by the SQLEE treatment. Additionally, inflammation-related signaling pathways, including mitogen-activated protein kinase (MAPK) signaling and NF-κB activation, have been studied. MAPKs are involved in the production of inflammatory mediators, including TNF-α and COX-2. The NF-κB induces inflammation not only directly by increasing the production of inflammatory cytokines and chemokines but also by regulating cell proliferation, apoptosis, and differentiation. NF-κB and MAPK signaling pathways are the major pathways associated with inflammation, which can be targeted in different inflammatory (UC and Crohn’s disease) and inflammation-associated diseases, such as cancer, Alzheimer’s disease, and obesity [45]. In the MAPK signaling pathway, the phosphorylation of ERK, JNK, and p38 was enhanced in the DS treatment group and was significantly suppressed by the SQLEE treatment. Similarly, IκB phosphorylation was enhanced by the DS treatment but suppressed by the SQLEE treatment. Suppression of IκB phosphorylation can suppress the activation of the NF-κB pathway, a key inflammatory signaling pathway. Suppression of both NF-κB and MAPK signaling pathways by the SLQE strengthened its anti-inflammatory potential (Figure 3). The study showed that SQLEE exhibits anti-UC activity by suppressing inflammatory signaling pathways and subsequently inhibiting inflammatory mediators [44].

In a later study, the researchers reported anti-UC activity due to the antioxidant effects of SQLEE. Similar to the previous study, two doses (100 and 300 mg/kg) of SQLEE were administered in a mouse model of DS-induced colitis. High-dose SQLEE significantly suppressed the DAI score, exhibiting a stronger effect than the positive control drug (sulfasalazine). Histopathological analysis of the colon tissues revealed the potential therapeutic effect of SQLEE through the suppression of tissue damage and crypt distortion. SQLEE treatment produced an insignificant improvement in whole gut transit time (gut motility), which had been significantly reduced by DS. 8-Oxo-dG-based evaluation revealed increased DNA damage in the colon tissues. However, high-dose SQLEE treatment significantly reduced the 8-oxo-dG-positive cells in the colon tissue, suggesting the suppression of DNA damage. Plasma malondialdehyde (MDA) levels, which had been enhanced by DS administration, were significantly suppressed by the antioxidant effect of SQLEE. SQLEE treatment significantly increased the plasma SOD and catalase activities in the colon, which were suppressed in the DS treatment group. The expression of antioxidant enzymes such as SOD1, SOD2, and GPx, as analyzed by WB, revealed that SQLEE treatment significantly increased SOD1 expression, which was suppressed in the DS treatment group. However, SOD2 and GPx expressions were significantly suppressed by the SQLEE treatment, as indicated by their higher levels in the SD treatment group.

The role of SQLEE in the gut microbiome and its anti-UC activity were studied in a mouse model of DS-induced colitis. Notably, in these studies, the anti-inflammatory compounds tricin (820 µg/g) and *p*-coumaric acid (1130 µg/g) were the only ones quantified in the SQLEE and considered important components for the anti-UC activity [44,46]. Similar to earlier studies, the incidence of diarrhea and bloody stools was increased in the DS group, which in turn increased the DAI score. However, the DAI score was significantly suppressed in the treatment groups. Similarly, the DS group showed a reduction in the mean colon length, which was significantly restored by SQLEE treatment. Gut microbiome analysis revealed the suppression of OTUs, the Chao1 estimator, and the Shannon diversity index following DS administration. These parameters were restored by the SQLEE treatment. Compared with the control, DS increased the ratio of Bacteroidetes to Firmicutes. In this study, the ratio of Bacteroidetes to Firmicutes was restored by the SQLEE treatment. Collectively, these results suggest that gut dysbiosis in DS-induced UC was restored by the SQLEE treatment.

### 5.7. Anti-Cancer Activity

The incidence of metabolic diseases such as obesity, diabetes, and cancer is increasing globally. Side effects and drug resistance are important factors that hinder the effectiveness and long-term use of anticancer drugs. Phytochemicals have shown potential against various cancers [47], and they are considered safer than harsh chemotherapies [48]. The anticancer activity of SQLE has been analyzed in various in vitro and animal experiments.

#### 5.7.1. In Vitro Anticancer Activity of *S. quelpaertensis* Extracts

Initially, *S. quelpaertensis* was studied for its anticancer activity, owing to its anti-inflammatory applications in traditional medicine. In an early study, the anticancer potential of the leaves of *S. quelpaertensis* was evaluated in human leukemia cells (HL-60) [22]. The methanol extract of the leaves and their fractions in hexane, butanol, water, and ethyl acetate were evaluated for their anticancer activities. The fractions of the leaf extract showed a significant inhibitory activity. The activity of the ethyl acetate fraction was slightly higher than that of the positive control (epigallocatechin gallate) used in this study. Detection of annexin-V and other apoptosis-related parameters using flow cytometry indicated that the extract induced apoptosis through increased nuclear condensation. WB analysis showed that the expression of cleaved caspases 3 and 9 increased and PARP degradation occurred in a dose-dependent manner. These findings further support the observation that apoptosis increased following extract treatment [22].

In another study, after the leaf extracts were shown to exert an effective antioxidant action according to different methods, the MTT assay was performed to evaluate the anticancer activity of leaf extracts. The extracts were prepared using ethanol and water solvents to study anticancer activity in the human colon cancer cell line (HCT116). Both water and ethanol extracts showed significant dose-dependent inhibition of HCT116 cell growth. Similar to the antioxidant activity, the anticancer activity was higher in the ethanol extract than in the water extract, which corroborates the higher content of TP and TF in the ethanol extract than in the water extract [23].

Lung cancer is associated with drug resistance. As combination therapy may serve as an important approach against lung cancer, the leaf extract of *S. quelpaertensis* was evaluated alone and in combination with cis-diammineplatinum II (CDP), a drug used to treat various cancers, including lung cancer. The anticancer activity was studied using the lung cancer cell lines A549 and H1299 [24]. According to MTT assays, *S. quelpaertensis* alone and in combination with CDP significantly inhibited both cell lines. WB analysis was used to study the role of the important signaling pathway phosphoinositide-3 kinase (PI3K)/protein kinase B (AKT)/mammalian target of rapamycin (mTOR), which is involved in cell proliferation, tumorigenesis, cell invasion, and drug resistance. The WB analysis showed that the phosphorylation of PI3K and mTOR was significantly suppressed by *S. quelpaertensis* treatment, suggesting that the PI3K-AKT-mTOR signaling pathway plays a role in the anticancer activity of *S. quelpaertensis* [24]. The PI3K/AKT/mTOR axis is the most frequently activated signaling pathway in cancer and is often associated with drug resistance [49]. PI3K/AKT/mTOR inhibitors have shown promising results in clinical studies [50].

Furthermore, the suppression of cancer stem cells (CSC), which contribute to self-renewal, high proliferation, and chemoresistance, was analyzed using CD44 and SOX-2 markers. Flow cytometry-based analysis revealed the suppression of the CSC population by *S. quelpaertensis* treatment; this effect was higher with the combination of *S. quelpaertensis* and CDP. Furthermore, the self-renewal capacity of the CSC was studied in H1299 spherical cultures exposed to the treatment. Dose-dependent inhibition of the size and number of spheres was observed in both *S. quelpaertensis* and the combination treatments. Similarly, the colony formation of CSCs was significantly suppressed by *S. quelpaertensis* alone and in combination with CDP. The effects of *S. quelpaertensis* alone and in combination with CDP on the metastasis of lung cancer cells were studied using wound-healing and invasion assays. In both assays, *S. quelpaertensis* treatment suppressed cell migration; this effect was higher with the combination (*S. quelpaertensis* with CDP) treatment. Additionally, gelatin zymography was used to evaluate the effects of *S. quelpaertensis* on the activity of matrix metalloproteinases (MMPs), which are a family of zinc-dependent endopeptidases. MMPs degrade matrix proteins such as type IV collagen and fibronectin, which facilitate the invasion of cancer cells and metastasis. WB and zymography assays showed that *S. quelpaertensis* treatment suppressed both expression and activity of MMPs, respectively; however, this effect was higher with the combination (*S. quelpaertensis* with CDP) treatment [24].

In another study, the anticancer activity of *S. quelpaertensis* and its major compounds present in the extract were evaluated in the colon cancer cell lines HT29 and HCT116 [13]. For this purpose, CD133+ and CD44+-stained CSCs isolated from HT29 and HCT116 cells were used. In the case of both cell lines, flow cytometry-based analysis revealed that 200 and 300 μg/mL SQLEE significantly suppressed colony formation of CSCs. Another widely used self-renewal assay, the sphere-formation assay, revealed a significant suppression in the number of spheres upon treatment with SQLEE. Furthermore, Wnt/β-catenin signaling, which is important for the formation of CSCs, was studied to explore the possible mechanism by which SQLEE is involved in CSC suppression. According to WB analysis, SQLEE suppressed the expressions of β-catenin in the cytosol and nuclear region in both cell lines, with *S. quelpaertensis* exhibiting the greatest effect. Phosphorylation of GSK3β at the Ser9 position decreases its activity in ubiquitin-targeted degradation of β-catenin. According to WB analysis, SQLEE significantly reduced the phosphorylation of GSK3β, with SQLEE showing a higher effect. These results suggested that the SQLEE treatment suppressed the Wnt/β-catenin signaling through suppression of β-catenin level by inhibiting its ubiquitin-targeted degradation. Additionally, the effect of SQLEE on CSC differentiation was studied, as it is an important characteristic of CSCs. The differentiation marker CK20 was enhanced in both cell lines, following SQLEE treatment. Additionally, an RT-PCR study showed that SQLEE treatment suppressed the expression of stem cell markers such as CD133, CD44, DLK1, Notch1, Sox-2, and VEGF [13]. SQLEE treatment significantly suppressed clonogenic capacity and Notch1 expression.

In a later study, human cancer cell lines, including SNU-1, SNU-16, HL-60, MKN-45, and MKN-74, were used to evaluate the anticancer potential of *S. quelpaertensis* against various cancers [4]. The phytochemical-rich extract of *S. quelpaertensis* leaves (PCREL) was prepared as described in a previous study [51], and its fractions were obtained using different solvents such as butanol, chloroform, ethyl acetate, and hexane. On the basis of its antiproliferative activity in cancer cell lines and non-cytotoxicity in normal cells, the ethyl acetate fraction of the extract was further evaluated in this study. Growth inhibition with PCREL was observed in all cell lines, in the following order: MKN-74 cells (81%) > SNU-16 cells (80%) > HL-60 cells (60%) > MKN-45 cells (51%) > SNU-1 cells (30%) [4]. The induction of apoptosis was studied to explore the possible mechanism behind the anticancer activity of the extract, which was evaluated using flow cytometry. The treated cells showed a dose-dependent induction of apoptosis and an increase in G1 arrest. Furthermore, the molecular mechanism underlying apoptosis was investigated using WB analysis to analyze the expression of apoptosis-related proteins, such as Bax, Bcl-2, procapspase-3, and PARP. Both HL-60 and MKN-74 cells showed suppression of the anti-apoptotic protein procapspase-3, PARP, and Bcl-2, and increased expression of the apoptosis-promoting Bax protein [4].

The mechanism behind the anticancer effects of *S. quelpaertensis* was evaluated in wild-type p53 (p53-WT) and p53 knockout (p53-null) cells of human colon carcinoma (HCT116). The anti-proliferative activity of the ethanol extract of the leaves was evaluated using the trypan blue exclusion assay. The extract suppressed the proliferation of both p53-WT and p53-null HCT116 cells in a time- and dose-dependent manner [25]. Furthermore, the Annexin V apoptotic assay revealed a time-dependent increase in the number of apoptotic cells in both p53-null and p53 wild-type cells used in the study. Cell cycle analysis showed an increase in the percentage of cells in the S and G2/M phases, whereas that of cells in the G1 phase decreased after treatment. A time-dependent increase in NO production and the expression of three isoforms of NOS (eNOS, iNOS, and nNOS) was observed following the treatment with the extract. The mechanism of cell death was studied by evaluating the expression of the inhibitors of apoptosis (IAP) family using RT-PCR and WB analysis. The treatment suppressed the gene expressions of CIAP-1 and CIAP-2 in a time-dependent manner, according to the RT-PCR analysis, and the protein expressions of survivin, CIAP-1, and CIAP-2. The treatment did not alter the gene and protein expressions of XIAP, according to the RT-PCR and WB analyses, respectively. Furthermore, the extract treatment suppressed bcl-2 and enhanced PARP expression according to WB analysis, whereas it increased caspase 3 activity by 2-fold. The study suggested that the suppression of the anti-apoptotic protein bcl-2 and apoptotic regulatory proteins such as survivin, CIAP-1, and CIAP-2 may contribute to the induction of apoptosis. These results support the earlier finding of apoptosis induced by SQLE in human leukemia HL-60.

#### 5.7.2. In Vitro Anticancer Activity of Compounds

In a previous study, the anticancer activities of SQLE and its two major constituents, tricin (0.7 μM) and *p*-coumaric acid (1.8 μM), were evaluated against the HT29 and HCT116 colon cancer cell lines (at concentrations equivalent to 300 μg/mL of extract) [13]. Flow cytometry-based analysis revealed that tricin and *p*-coumaric acid suppressed CSC colony formation; however, the effect was not statistically significant. As assessed by the sphere-formation assay, both tricin and *p*-coumaric acid significantly inhibited the self-renewal capacity of the cells, evidenced by a decrease in sphere count. Furthermore, tricin and *p*-coumaric acid suppressed the expression of β-catenin in the cytosol and nuclear region in both cell lines. Tricin significantly reduced the phosphorylation of GSK3β. In HT29 cells, *p*-coumaric acid was found to specifically enhance the levels of the CK20 marker during CSC differentiation. Additionally, tricin and *p*-coumaric acid suppressed the expression of stem cell markers such as CD133, CD44, DLK1, Notch1, Sox-2, and VEGF [13]. These results clearly show that the major bioactive compounds suppress CD133+CD44+-positive HT29 and HCT116 cells [13]. A clonogenic assay was performed to determine the effect of the combination of *p*-coumaric acid and tricin on clonogenicity by measuring the expression of the stem cell marker Notch1. SQLE treatment significantly suppressed clonogenic capacity and Notch1 expression, exhibiting a higher effect than *p*-coumaric acid, tricin, and the combination of *p*-coumaric acid and tricin. Since *p*-coumaric acid and tricin were tested at concentrations equivalent to those found in the extract, these results suggest that the anticancer activities of SQLE may be attributed not only to tricin and *p*-coumaric acid but also to other compounds present in the extract.

In an earlier study, the main component of SQLE, *p*-coumaric acid, was also studied for anticancer activity against multiple human cancer cell lines, including SNU-1, SNU-16, HL-60, MKN-45, and MKN-74 [4]. The *p*-coumaric acid inhibited all cell lines used in this study, in the following order: HL-60 cells (88%) > SNU-16 cells (48%) > SNU-1 cells (40%) > MKN-74 cells (25%) > MKN-45 cells (21%). Furthermore, the *p*-coumaric-treated cells showed a dose-dependent induction of apoptosis and an increase in G1 arrest. Consistent with the effects of SQLE, *p*-coumaric acid treatment in HL-60 and MKN-74 cells led to the downregulation of the anti-apoptotic protein Bcl-2, as well as procaspase-3 and PARP, while concurrently upregulating the pro-apoptotic protein Bax [4].

#### 5.7.3. In Vivo Anticancer Activity of *S. quelpaertensis* Extracts

Inspired by the in vitro studies, the anticancer activity of SQLEE was evaluated using an animal model. Male BALB/c nude mice were used to create a tumor xenograft model by subcutaneous injection of CD133+ and CD44+ double-stained HT29 cells. After nine weeks of treatment with SQLEE, the tumor volume decreased by 40.1% compared to that of the control, which was not significant. Furthermore, the expression of CSC markers, such as CD133, CD44, DLK1, Notch1, and Sox-2, was suppressed by SQLEE treatment. Additionally, suppression of β-catenin expression and GSK3β phosphorylation at the Ser9 position suggested the suppression of Wnt/β-catenin signaling in the treatment group. In most tumors, the WNT/β-catenin signaling pathway is often abnormally activated. The WNT/β-catenin signaling pathway plays important roles in tumorigenesis, metastasis, and drug resistance [52]. Hypoxia-inducible factor-1α (HIF-1α) signaling is important in hypoxic tumors, which express certain CSC markers and are considered highly tumorigenic. The SQLEE treatment group showed suppression of the expression of HIF-1α and its downstream target VEGF compared with the control group. These results, especially the suppression of DLK1, Sox-2, CD133, Notch1, HIF-1α, β-catenin, VEGF, and CD44, and the phosphorylation of GSK3β, are consistent with the in vitro results of the extract in HT29 cells. This suggests that the extract’s bioactivity can be successfully recapitulated under in vivo conditions. Similar to the anti-UC activity, the anti-inflammatory compounds tricin and *p*-coumaric acid present in SQLEE were considered active constituents important for its anticancer activity. However, the higher activity of SQLEE compared to that of the individual compounds highlights the importance of phytoconstituent profiling for optimizing SQLEE’s effects.

### 5.8. Anti-Depressant Activity of S. quelpaertensis Extracts

Depression is a major mental health disability that is expensive to treat and common, and it increases the risk of suicide. Adverse situations, such as other disease conditions, can cause or worsen depression [53,54,55].

Several phytochemicals have shown potential in the treatment of depression [56]. Thus, the antidepressant activity of the leaf extract of *S. quelpaertensis* was studied in an ovariectomized rat model of depression [8]. SQLE was orally administered to ovariectomized female Sprague Dawley (SD) rats for 14 consecutive days.

The two-week stress period induced depression symptoms characterized by immobility for a longer time compared to that in the normal group, according to the tail suspension test. Low-dose SQLE (100 mg/kg) significantly reduced the immobility time. Similarly, low-dose SQLE significantly reduced the immobility time in the forced swimming test, compared with the control treatment.

The cutaneous body temperature of the rats increased significantly after ovariectomy compared with that in the normal group. The SQLE-administered groups showed a significant decrease in cutaneous body temperature on the last day of treatment. Treatment with low-dose SQLE significantly increased the serotonin levels in the prefrontal cortex (PFC) and hypothalamic regions of the brain and the dopamine levels in different areas of the brain, compared with the control. TH-positive cells were suppressed in the brains of ovariectomized animals, which were restored following the 14-day low-dose SQLE treatment. Similarly, hypothalamic TPH+ cells were diminished in the brains of ovariectomized animals, which was restored by 14-day low-dose SQLE treatment in this study. Additionally, the expression of TPH-IR cells in the peduncular part of the lateral hypothalamus and the expression of PKC-IR cells in the hypothalamic area were remarkably suppressed in the ovariectomized group compared to the normal group. In this experiment, the expression of both cell types was restored by a 14-day low-dose SQLE treatment. In summary, the study suggested that the antidepressant effect observed in both behavioral tests may be due to an increase in the neurotransmitters dopamine and serotonin in different parts of the brain. The increase in cellular expression supports an increase in the levels of these neurotransmitters. Conversely, a high dose of SQLE did not provide a significant antidepressant effect, emphasizing the need for further research to elucidate the mechanism and components of SQLE responsible for its activity [8].

### 5.9. Anti-Fatigue Activity S. quelpaertensis Extracts

Chronic fatigue is prevalent in the general population and is expected to increase because of rapid urbanization and stress in competitive cultures [57]. An estimated 54% reduction in workforce productivity occurs because of untreated chronic fatigue syndrome in the United States [58]. Several plant extracts have been used to develop anti-fatigue interventions because of their antioxidant and anti-inflammatory properties. SQLE has been studied for its anti-fatigue activity in male ICR mice [59]. In a 7-day exhaustive swimming test, the average swimming time was significantly longer in the SQLE-administered group than in the exercise control group. Clearly, the increase in the exercise endurance capacity of the mice demonstrated the anti-fatigue properties of SQLE. Thus, the effect of SQLE on the metabolic parameters associated with fatigue, such as the levels of blood glucose and lactate, and levels of muscular lactate and glycogen, was studied. Blood glucose and muscle glycogen levels were significantly higher in the SQLE group than in the control group. However, blood and muscle lactate levels were significantly lower in the SQLE group than in the control group. These results suggest that the anti-fatigue properties of SQLE are linked to an improvement in the metabolites associated with fatigue. Adenosine monophosphate-activated protein kinase (AMPK) signaling, which is associated with protein metabolism, was studied using WB analysis in L6 skeletal muscle cells cultured under hypoxic conditions. Hypoxia-induced expression of phosphorylated AMPK, phosphorylated acetyl-CoA carboxylase, and GLUT4 was suppressed by SQLE treatment. Whole transcriptome analysis can provide a comprehensive molecular mechanism underlying treatment [60]. Hence, to understand the comprehensive molecular mechanism of the anti-fatigue property of SQLE, comparative RNA sequencing of the muscular transcriptome was conducted for the SQLE and exercise control groups. A total of 835 genes were differentially expressed between the SQLE and exercise control groups. These differentially expressed genes were enriched in biological processes, such as glycogen metabolism, lipid metabolism, carbohydrate metabolism, ROS metabolism, and transport. Similarly, these DEGs were enriched in AMPK signaling, insulin signaling, carbon metabolism, endocytosis, and cytokine–cytokine receptor interaction. This suggested that SQLE exerts significant anti-fatigue activity through the improvement of fatigue-related metabolites, which may be associated with the modulation of AMPK signaling.

### 5.10. Anti-Diabetic Activity of S. quelpaertensis Extracts

Considering the different pharmacological activities of *S. quelpaertensis*, the anti-diabetic activity of *S. quelpaertensis* leaves was evaluated in an in vitro study using L6 rat skeletal muscle cells [31]. In the anti-diabetic study, the major components of SQLE, tricin (7430 µg/g) and *p*-coumaric acid (2690 µg/g), were quantified. These compounds are believed to be important for the anti-diabetic activity, as they are known to have antioxidant and anti-inflammatory properties that can support the anti-diabetic effect. Glucose uptake and lipid metabolism in L6 muscle cells were analyzed using a 2-[*N*-(7-nitrobenz-2-oxa-1,3-diazol-4-yl) amino]-2-deoxyglucose (2-NBDG) uptake assay and triglyceride accumulation, respectively. Glucose uptake by the L6 muscle cells increased in a dose-dependent manner after SQLE treatment. Similarly, AMPK phosphorylation showed a dose-dependent increase following SQLE treatment, which suggested that the AMPK signaling pathway plays a role in SQLE-mediated glucose uptake. The expressions of CD36, fatty acid translocase (FAT), and transcription factor PPARγ, which are associated with lipid uptake, were increased with SQLE treatment. Triglyceride accumulation in L6 cells treated with oleic acid was significantly suppressed by SQLE. Additionally, SQLE suppressed the expression of sterol regulatory element-binding protein (SREBP)-1c and fatty acid synthase (FAS). This study suggested that an increase in acetyl-CoA carboxylase (ACC) phosphorylation might contribute to the suppression of oleic acid-induced triglyceride accumulation. In summary, this study concluded that the antidiabetic activity of SQLE may be due to the activation of AMPK pathways. A major limitation of in vitro assays is their inability to simulate the multi-organ complexity of diabetes. Unlike in vivo models, they do not consider bioavailability or the intricate hormonal and neural pathways involving the liver, pancreas, and peripheral tissues. Further in vivo studies are required to establish the antidiabetic activity of SQLE.

### 5.11. Anti-Obesity Activity

*S. quelpaertensis,* a bamboo grass, has been used in traditional medicine because of its various pharmacological activities. However, its health-promoting effects on lipid metabolism, which may be important for exerting an anti-obesity effect, have not been effectively studied. Nevertheless, researchers have evaluated the anti-obesity effects of *S. quelpaertensis* in both in vitro and in vivo studies.

#### 5.11.1. In Vivo Anti-Obesity Effect of *S. quelpaertensis* Extracts

The anti-obesity effect of SQLE was evaluated in C57BL/6 mice fed a high-fat diet (HFD) [5]. In the HPLC-based phytochemical profiling, four compounds were identified in the SQLE: *p*-coumaric acid (23,706 µg/g), tricin (28 µg/g), chlorogenic acid, and isoorientin. Clearly, among these constituent phytochemicals, *p*-coumaric acid was the major compound and is thought to be important for the observed activity. The HFD-induced increase in body weight was significantly suppressed by SQLE. Similarly, the increase in the weights of epididymal and perirenal adipose tissues due to the HFD was significantly suppressed by SQLE treatment. Histological analysis showed an increase in the size of epididymal adipocytes due to the HFD, which was significantly decreased by SQLE treatment. Additionally, the levels of total cholesterol (TC) and triglycerides (TG) in the serum, which had increased in the HFD group, were significantly suppressed by the treatment.

Serum levels of cell damage markers, including glutamic oxaloacetic acid transaminase (GOT), lactic dehydrogenase (LDH), and glutamic-pyruvic acid transaminase (GPT), were significantly increased by HFD but were significantly decreased by the SQLE treatment. In the liver tissues, the accumulation of lipid droplets was observed through HE staining in the HFD group, which was diminished by the SQLE treatment. Additionally, activation of the AMPK signaling pathway was studied in the study. The AMPK signaling pathway is associated with anti-obesity effects, as AMPK activation can promote tissue-specific lipolysis, energy expenditure in adipose tissue, and inhibit fatty acid synthesis by phosphorylating ACC to decrease the malonyl-CoA level [61]. In the study, the expression of phosphorylated AMPK and ACC in epididymal adipose tissues, which was suppressed by the HFD, was significantly increased by the SQLE treatment. Furthermore, the gene expression of adiponectin in the epididymal adipose tissue was suppressed by the HFD but significantly restored by the SQLE treatment. Activation of the AMPK pathway may be a major factor behind the anti-obesity effect of SQLE observed in the study.

Subsequently, a study was conducted on male SD rats to study the effects of two different doses of SQLE supplements (5% and 3%). After an 11-week treatment with 5% SQLE, the levels of TC, TG, low-density lipoprotein cholesterol (LDL-C), and atherogenic index (AI) values, which had been increased by HFD, were significantly suppressed by the SQLE treatment [62]. Conversely, the HDL-C level, which was suppressed by the HFD, significantly increased with SQLE treatment. An increase in plasma adiponectin levels was observed in the treatment group; however, this increase was not significant. However, 5% SQLE significantly suppressed plasma resistin levels, which had been increased by the HFD. Increased expression of resistin in the heart is associated with the worsening of cardiovascular metabolic symptoms. The gene expression level of resistin in the heart, which had been increased by the HFD, was significantly suppressed by the 5% SQLE treatment. Furthermore, the 5% SQLE treatment suppressed the gene expression of PPAR, C/EBPb, aP2, and SREBP-1 in visceral adipose tissue; however, only C/EBPb showed a significant difference. Histological analysis revealed HFD-induced steatosis and large fat droplets in rat livers. Treatment with 5% SQLE decreased the size of fat droplets, infiltration of inflammatory cells, and swelling of hepatic cells in a dose-dependent manner. In liver lipid profiling, hepatic TG and total lipid levels, which had been increased by the HFD, were significantly suppressed by the SQLE treatment. The molecular mechanism of SQLE in regulating the lipid profile in the liver was studied, and both the protein and gene expression of FAS and SREBP-1, which had been increased by the HFD, were significantly suppressed by SQLE treatment. Similarly, the expression of UCP-2, which is associated with thermogenesis and lipid metabolism, was increased by the HFD; however, it was significantly suppressed by the SQLE treatment [62].

#### 5.11.2. In Vitro Anti-Obesity Activity of *S. quelpaertensis* Extracts

To explore the role of AMPK in the anti-obesity effect of SQLE, the AMPK pathway was studied in 3T3L1 cells. Mature 3T3-L1 adipocytes were subjected to various concentrations of SQLE, which produced a dose-dependent increase in the phosphorylation of both AMPK and ACC, consistent with the findings of the in vivo study. Additionally, SQLE treatment increased the expression of the fatty acid oxidation gene CPT-1a [5].

In an in vitro study, the anti-obesity effects of SQLE and its main constituents were studied in 3T3-L1 cells [32]. SQLE inhibited cellular lipid accumulation, as indicated by staining with Oil Red O dye, in a dose-dependent manner, showing 83% inhibition at the highest dose used in the study. WB analysis showed that the protein expressions of PPARc, C/EBPa, SREBP-1c, and aP2 were suppressed by SQLE in a dose-dependent manner. The expressions of FAS and adiponectin were suppressed by SQLE treatment in RT-PCR experiments. The AMPK pathway plays a key role in the expression of obesity-related genes such as SREBP-1c. WB analysis revealed that SQLE treatment increased AMPK and ACC phosphorylation in a dose-dependent manner during the early phase of differentiation. The expression of SREBP-1c decreased 12 h after the induction of differentiation. These findings suggested that SQLE suppressed adipogenesis via AMPK signaling during the early phase of differentiation [32]. The suppression of FAS and the activation of AMPK signaling via increased phosphorylation of AMPK and ACC were also observed in the in vivo experiments. This supports the reduction in lipid accumulation observed in both the cell and animal models. Molecular targets that are consistently modulated in both in vitro and in vivo experiments should be prioritized as key candidates for future investigation.

#### 5.11.3. In Vitro Anti-Obesity Activity Compounds

*p*-Coumaric acid, the main bioactive constituent of SQLE, was also tested on 3T3-L1 cells to evaluate its individual contribution to the extract’s activity. The application of *p*-coumaric acid decreased lipid accumulation and the expression of transcription factors (C/EBPa and SREBP-1c) during the late stage of adipocyte differentiation. In fully differentiated 3T3-L1 adipocytes, *p*-coumaric acid significantly enhanced AMPK and ACC phosphorylation, as indicated by WB analysis, and enhanced the mRNA expression of CPT-1a. In summary, the findings suggested that SQLE exerts its anti-obesity effects by suppressing the transcription factors (C/EBPa and SREBP-1c) through the AMPK signaling pathway in the initial phase of adipogenesis [32]. Importantly, significant phosphorylation of AMPK and ACC was observed with *p*-coumaric acid at 12.5–25 µg/mL and higher, matching the concentration found in the extract and supporting its contribution to the anti-obesity activity. Notably, the extract affected the initial phase of adipogenesis effectively, while *p*-coumaric acid was most effective at a later stage [32]. This difference in timing suggests that other constituents in the extract may act synergistically to inhibit the early stages of differentiation.

### 5.12. Hepatoprotective Effects

The liver is a vital organ with a wide range of functions that are essential for overall health, and its malfunction can severely affect other important organs [63]. The hepatoprotective activity of SQLE was evaluated in in vitro and in vivo experiments.

#### 5.12.1. In Vitro Hepatoprotective Effects of *S. quelpaertensis* Extracts

After demonstrating the hepatoprotective activity of SQLE in mice, the effect of SQLE and its main constituent (*p*-coumaric acid) on lipid metabolism and the role of the AMPK pathway in HepG2 cells were investigated. The AMPK signaling pathway can suppress lipid accumulation, which is the main reason behind liver steatosis, and natural herbs are reported to protect the liver through activating the AMPK pathway [64]. The study utilized a liver steatosis model, in which HepG2 cells exposed to oleic acid exhibited intracellular lipid accumulation [33]. Intracellular lipid accumulation, which had been increased by the oleic acid exposure, was significantly suppressed by the SQLE and *p*-coumaric acid treatments. Similarly, according to WB analysis, both treatments (SQLE and *p*-coumaric acid) significantly enhanced AMPK and ACC phosphorylation, which had been induced by liver steatosis. Additionally, the expressions of SREBP-1c and FAS, which had been increased because of liver steatosis, were significantly suppressed by SQLE treatment. These results suggested that the hepatoprotective activity of SQLE and *p*-coumaric acid may be attributed to the activation of the AMPK pathway, which suppresses intracellular lipid accumulation [33].

In a previous study, the hepatoprotective effects of SQLE against ethanol-induced toxicity were examined in HepG2 cells. The administration of 400 mg of ethanol significantly suppressed the viability of HepG2 cells; 60% ethanol SQLEE exhibited a pattern of increasing the viable cell percentage in the ethanol-exposed cells [34].

The hepatoprotective activities of SQLEE in different solvent fractions (*n*-hexane, chloroform, ethyl acetate, *n*-butanol, water-saturated butanol, and distilled water) were studied in HepG2 cells subjected to alcohol-induced toxicity [20]. MTT assay showed that SQLEEEA effectively increased the viability of HepG2 cells exposed to ethyl alcohol. The HPLC-based identification of phytochemicals in SQLEE fractions revealed the presence of 14 flavonoids (Table 1). The SQLEEEA fraction contained a high content of flavonoids (152.23 µg/g of extract), benzoic acid derivatives (32.9 µg/g of extract), and cinnamic acid derivatives (21.7 µg/g of extract). The contents of *p*-coumaric acid and Myricetin were the highest among cinnamic acid derivatives and flavonoids, respectively (Table 1). Additionally, propidium iodide (PI) staining of HepG2 cells revealed that ethanol toxicity caused a two-fold increase in apoptotic DNA in the sub-G1 phase, which was reversed by SQLEEEA treatment. Similarly, the thymidine incorporation assay revealed that ethanol toxicity significantly inhibited HepG2 cell proliferation; this effect was significantly reversed by SQLEEEA treatment. This study demonstrates the hepatoprotective activity of SQLEEEA against ethanol toxicity [20].

Subsequently, alcohol-induced liver toxicity was studied in both in vitro and in vivo experiments [9]. Using MTT and colony-forming assays, the protective effects of SQLE prepared with different solvent concentrations were evaluated against ethanol toxicity in HepG2 cells, which was induced using a high concentration (800 mM) of ethanol. SQLEE 80% protected against ethanol-induced toxicity in HepG2 cells. ROS and NO levels in HepG2 cells, which were elevated because of ethanol toxicity, were significantly suppressed by the SQLEE treatment. Additionally, the expression of catalase and GPX-1 was measured in this study; catalase expression, which had been suppressed by ethanol toxicity, was significantly enhanced by SQLEE treatment. GPX-1 expression was not significantly altered by ethanol treatment; however, its activity was significantly increased with SQLEE treatment. These findings suggested that the reduction of oxidants such as ROS may be due to the increased expression of antioxidant enzymes by SQLEE, which may contribute to its hepatoprotective activity [9]. Furthermore, animal studies supported the hepatoprotective and antioxidant activities of SQLEE.

#### 5.12.2. In Vitro Hepatoprotective Effects of Compounds

*p*-Coumaric acid, the main bioactive constituent of SQLE, was also studied for hepatoprotective activity to evaluate its individual contribution to the extract’s activity. The effect of *p*-coumaric acid on lipid metabolism and the role of the AMPK pathway in HepG2 cells were analyzed. The increase in intracellular lipid accumulation induced by oleic acid exposure was significantly suppressed by *p*-coumaric acid treatment. Similarly, *p*-coumaric acid treatment significantly enhanced the phosphorylation of AMPK and ACC, which had been suppressed by liver steatosis. These findings indicate that *p*-coumaric acid contributes to the hepatoprotective properties of SQLE by activating the AMPK pathway, thereby reducing intracellular lipid accumulation [33]. Notably, equivalent amounts of *p*-coumaric (up to 40 µg/mL) and the extract (up to 1000 µg/mL) exhibited concentration-dependent hepatoprotective effects in the HepG2 cells [33]. These effects included decreased lipid accumulation, the suppression of transcription factors, and the enhancement of AMPK and ACC phosphorylation. The correlated activities of the extract and *p*-coumaric support the conclusion that this compound is a key contributor to the extract’s hepatoprotective activities.

#### 5.12.3. In Vivo Hepatoprotective Activity of *S. quelpaertensis* Extract

In an initial study, the hepatoprotective effects of SQLEE against ethanol toxicity were evaluated in female C57BL/6 mice [65]. Ethanol toxicity was induced using three intraperitoneal doses of 30% ethanol at 12 h intervals. SQLE (100 mg/kg BW) significantly decreased the serum activity of liver damage markers, such as aspartate aminotransferase (AST) and alanine aminotransferase (ALT), which had been markedly increased by ethanol toxicity. Lipid peroxidation in the liver tissue, which was increased because of ethanol toxicity, was significantly decreased by SQLEE treatment. A histological study of liver tissues supported the protective effect of SQLEE, as ethanol-induced signs of liver steatosis, such as increased lipid vacuoles and loss of membrane integrity, were alleviated by SQLEE treatment [65]. Different fractions (butanol, methylene chloride, and water) of SQLEE were studied for their hepatoprotective activities. Among the fractions, only the butanol fraction showed protective activity by suppressing lipid peroxidation, AST and ALT levels, and GSH depletion in tissues [65].

After analyzing the liver-protective activity of SQLE in an in vitro cell line study, two doses (10 mg and 100 mg/kg BW) of 80% ethanol SQLEE were evaluated for their hepatoprotective activity against ethanol-induced hepatotoxicity in a binge model of alcohol consumption. The alcohol content in the serum, which had been elevated by ethanol administration, was reduced by more than 50% after SQLEE treatment. Histopathological analysis of liver tissues revealed steatosis with an increase in lipid droplets, ballooning degeneration, and loss of cellular boundaries due to ethanol toxicity. Dose-dependent recovery of steatosis symptoms was observed following SQLEE treatment. In the TBARS assay, lipid peroxidation in the liver tissue, which had increased because of ethanol toxicity, decreased significantly with SQLEE treatment. Expression of the antioxidant GSH in the liver, which had been suppressed by ethanol toxicity, increased significantly with SQLEE treatment. Immunohistochemical analysis showed that CYP2E1 expression in the ethanol-exposed liver, which had increased owing to ethanol toxicity, decreased significantly with SQLEE treatment. Additionally, WB analysis was used to validate CYP2E1 expression, and it showed similar results. The study suggested the potential therapeutic effectiveness of SQLEE against ethanol toxicity [9].

In another study, the hepatoprotective activities of SQLEE were evaluated through in vivo experiments following phytochemical component analysis and in vitro studies on hepatoprotective activities. Three doses (10, 50, and 100 mg/kg BW) of SQLEE were studied in animal models, with normal (untreated) and negative controls (only ethanol) [20]. The appearance of healthy livers from the normal control group was bright red, whereas that of livers from animals with ethanol toxicity was pale. SQLEE treatment restored liver appearance in a dose-dependent manner. Similarly, a histopathological study revealed an increase in lipid droplets, ballooning degeneration, and the loss of cellular boundaries due to ethanol toxicity. Dose-dependent recovery of steatosis symptoms was observed following SQLEE treatment. Similar to a previous study, an improvement in CYP2E1 expression was also observed through WB and immunohistochemical analyses. Similarly, the suppression of lipid peroxidation and GSH level was reversed by SQLEE treatment, as observed in TBARS and glutathione colorimetric assays, respectively. In WB analysis, SQLEEEA treatment produced a dose-dependent enhancement of AMPK phosphorylation in the liver, which had been suppressed by ethanol toxicity. Ethanol-induced toxicity increased the expression of the target enzyme of AMPK, FAS, which is associated with fatty acid synthesis; SQLEEEA treatment suppressed its expression in a dose-dependent manner. Additionally, the liver expression of perilipin-2 and TNF-α, associated with lipid accumulation, was increased because of the ethanol-induced toxicity; SQLEEEA suppressed these levels. It can be concluded that the antioxidant and anti-inflammatory properties contributed to the hepatoprotective activity of SQLEE, which was supported by both in vitro and animal studies. Similar to the anti-obesity results, the suppression of FAS and the activation of AMPK signaling via increased phosphorylation of AMPK and ACC were consistent in both the in vitro and in vivo experiments by the extract. This suggests a common mechanistic pathway supports the extract’s anti-obesity and hepatoprotective effects, demonstrating that these activities can be successfully recapitulated in vivo despite the inherent pharmacokinetic complexity of living systems.

#### 5.12.4. In Vivo Hepatoprotective Activity of Compounds

Following the demonstration of the SQLEEs’ hepatoprotective activity in an animal model, its primary phytochemical constituent, *p*-coumaric acid, was evaluated in the same model to identify the compound responsible for these effects [65]. In this study, *p*-coumaric acid demonstrated dose-dependent hepatoprotective activity against ethanol-induced toxicity by reducing lipid peroxidation, lowering serum AST and ALT levels, and preventing GSH depletion in liver tissues. The study suggests that *p*-coumaric acid is one of the primary active constituents responsible for the hepatoprotective activity of SQLEE [65].

### 5.13. Bone-Protective Activity

In a multifaceted study in an ovariectomized rat model (OVXR), the bone-protective activities of SQLE and green tea powder were analyzed, along with other parameters such as platelet aggregation, erythrocyte membrane Na channels, and lipid profiling of the plasma and liver. In this study, as the treatment, 10% *S. quelpaertensis* leaf powder (SQLP) was added to the diet of the OVXR. After four weeks of SQLP treatment, bone mineral density (BMD), bone mineral concentration (BMC), and bone width were measured in different groups of animals. Both BMD and BMC of the femur were significantly decreased in the OVXR group, which was partially recovered by SQLP treatment. The activity of the green tea powder was similar to that in another study [66]. This study highlighted the bone-protective effect of SQLP treatment on OVXR and suggested that further studies should be conducted to explore the possible mechanisms behind this effect.

### 5.14. Immunomodulatory Activity

Immunomodulatory activity enhances the overall defense of the organism, helping to fight infectious and noninfectious diseases. Therefore, herbal supplements or therapies that boost immunity can effectively protect against various diseases or conditions. Considering the importance of immune enhancement, the immunomodulatory activity of SQLE was studied in vitro and in vivo experiments.

#### 5.14.1. In Vitro Immunomodulatory Activity of *S. quelpaertensis* Extracts

The in vitro and in vivo immunomodulatory activities of SQLE and *Ficus erecta* (FE) were studied individually and in combination [35]. The in vitro studies were conducted in macrophages (RAW264.7 cells). The combination of SQLE and FE significantly increased the production of NO in these cells; the effect was the highest with the 2:8 combination ratio. The production of cytokines, such as IL-1β, IL-6, and TNF-α, in RAW264.7 cells was significantly increased in most of the treatment groups. The maximum increase in cytokine production was observed with the 2:8 combination treatment. These consistent results, indicating an increase in both NO production and cytokines, were further validated in an in vivo mouse model [35].

#### 5.14.2. In Vivo Immunomodulatory Activity of *S. quelpaertensis* Extracts

After achieving an effective immunomodulatory effect using the combination of SQLE and FE (2:8 ratio) in vitro, this combination was further studied in male BALB/c mice (Table 3). Three doses (50, 100, and 200 mg/kg) of the combination were evaluated for their immune enhancement activity in a 2-week study, which included control groups [35]. The cytotoxicity of natural killer cells was studied in splenocytes co-cultured with YAC-1 cells (target cells).

The combination treatment significantly increased the cytotoxicity of NK cells; this effect was greater than that of rutin, comparable to that of the positive control used in the study.

In the spleen, the production of cytokines, including IL-2, IL-4, IL-10, IL-12, and TNF-α, was significantly increased with the combination treatment.

In peritoneal exudate cells (PEC), the NO levels significantly increased after the combination treatment. The total number of cells at immunity-associated sites, such as the spleen, Peyer’s patches, draining lymph nodes (DLN), thymus, and PEC, was determined. The total number of cells showed a significant increase at all immunity-associated sites except the thymus, where the change in the total number of cells was insignificant.

Fluorescence-activated cell sorting (FACS) analysis of the spleen showed that the number of CD3+ T cells, CD49b+ NK cells, CD4+ Th cells, and CD8+ T cells significantly increased with the treatment compared to those in the control group. The significant increase in the expression level of CD4+/CD25+ Treg cells after the combination treatment suggests that the combination treatment increased the number of immune cells and regulated the immune system. The number of important B cell-related immune cells, such as CD23+/B220+ B cells, was increased by the treatment, suggesting the stimulation of both cellular and humoral immune responses. The number of Gr-1+/CD11b+ cells significantly increased following the combination treatment.

The activity of NK cells was studied based on the CD107a (LAMP-1) marker, which was significantly enhanced by the treatment.

Similar to the spleen, the DLN showed a significant increase in the number of CD3+ T cells, CD49b+ NK cells, CD4+ Th cells, and CD8+ T cells following the combination treatment. Furthermore, the number of CD4+/CD25+ Treg cells and CD23+/B220+ B cells was significantly enhanced by the treatment.

In the case of PEC, the number of CD3+/CD4+ Th cells, CD3+/CD8+ Tc lymphocytes, CD8+/CD25+ Treg cells, B220+/CD23+ B cells, B220+/CD69+ B cells, and CD11b+/CD69+ cells significantly increased with the treatment.

In the thymus, the number of CD4+ cells was significantly enhanced by the combination treatment at a high dose (200 mg/kg). In Peyer’s patches, the expression of CD3+, CD3+/CD4+ Th cells, and CD3+/CD8+ Tc lymphocytes was significantly enhanced by the combination treatment at a high dose (200 mg/kg). This study demonstrated immune enhancement in an animal model, which supported the potential of the combination as a therapeutic or supplement for immunomodulation.

The immunomodulatory effect of the fermented product of *S. quelpaertensis* leaves (SQLFP) was analyzed in another study. The immunomodulatory effects of SQLFP were studied in an animal model using positive (red ginseng) and negative (distilled water) controls. Fermentation of the leaves was performed using three different mushroom species, i.e., *Ganoderma lucidum* (SQLGL), *Phellinus linteus* (SQLPL), and *Hericium erinaceum* (SQLHE) [68]. β-glucans are considered important in immune enhancement; thus, their content was measured in all SQLFPs. The glucan content of the fermented product was the highest in SQLGL (7.40 ± 0.89 mg/100 mL), followed by SQLHE (4.49 ± 0.89 mg/100 mL) and SQLPL (3.73 ± 0.50 mg/100 mL). The T cell population in PBMCs after treatment showed a significant increase in the population of helper T cells, Th cells, and helper T cells and Th cells with cell-surface protein expression of CD4^+^CD8^−^ for all three fermentation conditions. Similarly, the macrophage population in the peritoneal cavity, which is important for innate immunity, was found to be significantly increased after SQLFP treatment, compared with that in the positive and negative control groups. Additionally, SQLFP significantly increased NO production in these macrophages compared with the negative control. The production of cytokines, such as IL-4, IL-10, and IFN-γ, in splenocytes significantly increased with the SQLFP treatment.

## 6. Discussion

Initial studies inspired by the use of *S. quelpaertensis* as a traditional medicinal and health drink led to the isolation and identification of phytochemicals with pharmacological activities. However, the presence of Pan Assay Interference Compounds (PAINS) in plant extract may impose limitations on the interpretation of pharmacological activity. Identification of phytochemical components of the extract, important for biological activities, may be challenging because the PAINS are the compounds that may give false positive results in the biological assays [69]. To date, analysis of SQLE for PAINS has not been reported in the literature. Thus, PAINS may be screened from the SQLE through wet lab identification and in silico prediction in future studies.

However, more than 30 important phytochemicals with important biological properties have been identified in SQLEs. These phytochemicals include 4-hydroxybenzoic acid, saikochromone A, isoorientin, daucosterol, lutein, *p*-hydroxybenzaldehyde, salicylic acid, syringaldehyde, methyl cis-*p*-hydroxycinnamate, methyl trans-*p*-hydroxycinnamate, *p*-coumaric acid, naringenin, vanillic acid, *N*-feruloylserotonin, *N*-*p*-coumaroylserotonin, and tricina and its derivatives (Table 1). Notably, the major compounds present in SQLE are not unique to *S. quelpaertensis*; they are also present in other plants. Nevertheless, the specific combination of these phytoconstituents may contribute to the multiple pharmacological activities of *S. quelpaertensis*. Information regarding the components important for the activity is crucial for the development of *S. quelpaertensis* as a therapeutic agent.

In the case of anti-tyrosinase activity, the compounds isolated from the extract clearly showed anti-tyrosinase activity, especially the serotonin derivatives. Thus, an optimal content of these compounds may enhance the anti-tyrosinase potency of SQLE.

The highest antioxidant activity was observed in the SQLE prepared from the May harvest, which also contained the highest levels of bioactive components, including TFC and TPC, as well as the compounds isoorientin and vanillic acid. The content of isoorientin is correlated with the antioxidant activity, and optimization of these components may improve the antioxidant potential of SQLE.

The HPLC-based method revealed the highest flavonoid content in the ethyl acetate fraction of SQLE compared to the fractions in other solvents, which also exhibited the highest hepatoprotective activity. The study also showed that SQLEEEA has the highest content of *p*-coumaric acid and Myricetin. The *p*-coumaric acid showed a hepatoprotective effect in the study. However, Myricetin isolated from SQLEEEA may also be studied for hepatoprotective activity in future studies.

In the case of anti-obesity activity, the HPLC-based phytochemical profiling of SQLE revealed that *p*-coumaric acid was the major phytochemical compared to the other phytochemicals quantified in the study. The phytochemical *p*-coumaric acid is thought to be important for the observed activity, but other phytochemicals, such as isoorientin, may also be important for the anti-obesity activity and should be studied in future research. For immune enhancement activity, only the content of β-glucans, a known immune enhancer, was estimated. Nevertheless, other *S. quelpaertensis* phytochemicals, such as flavonoids (Table 1), may also contribute to this activity and warrant further investigation.

In some important pharmacological activities, including anticancer, anti-diabetic, and anti-UC, only *p*-coumaric acid and tricin were quantified as considered important for these activities. However, the study showed that the anticancer activity of SQLE is higher than that of both of these compounds and even their combination. This suggests that other components of SQLE may also be important for its pharmacological activities. Thus, further studies are suggested to identify these other active phytochemicals in SQLE.

For several important pharmacological activities, such as antidepressant, anti-fatigue, and bone protection, studies are currently missing that identify the plant’s active constituents. We strongly suggest that these studies be conducted in the future.

*p*-Coumaric acid and tricin are the main compounds in *S. quelpaertensis* that are believed to be responsible for most of its biological properties. In subsequent studies, the extraction conditions were optimized to achieve high phenolic and flavonoid content, thereby maximizing the associated biological activities. Recent studies have analyzed seasonal variations in the phytochemical content of *S. quelpaertensis* to determine the optimal harvesting time of *S. quelpaertensis* for pharmacological usage [19]. Some of the main compounds of SQLE have been studied for their pharmacological activities and found to target major signaling pathways associated with cancer, obesity, liver toxicity, and inflammation.

Regarding anticancer activity, suppression of self-renewal capacity by *p*-coumaric acid and tricin may be attributed to Wnt/β-catenin signaling, as both compounds suppressed the expression of β-catenin in both the cytosol and nuclear regions [13]. Additionally, tricin treatment suppressed the phosphorylation of GSK3β, which is associated with the ubiquitin-targeted degradation of β-catenin [13]. Furthermore, the expression of stem cell markers (CD133, CD44, DLK1, Notch1, Sox-2, and VEGF) was suppressed by *p*-coumaric acid and tricin [13].

SQLE showed greater suppression of Wnt/β-catenin signaling and markers than the individual compounds, which suggested the role of other phytoconstituents in the anticancer activity of SQLE [13] (Table 3). The anticancer activity of *p*-coumaric acid was associated with the induction of apoptosis; thus, apoptosis through mitochondria-dependent pathway signaling was studied. The study revealed the enhancement of the pro-apoptotic gene Bax and downregulation of genes associated with apoptosis suppression, such as Bcl-2, procaspase-3, and PARP [4].

Regarding anti-inflammatory activity, *p*-coumaric acid and tricin suppressed LPS-induced enhancement of inflammation-related factors, including NO, IL-6, iNOS, and COX-2 [30]. Additionally, *p*-coumaric acid suppressed inflammation by targeting key inflammatory pathways, i.e., NF-κB signaling through suppression of phosphorylation and degradation of IκBα, which suppressed LPS-mediated activation of NF-κB [30]. Additionally, it suppressed the TNF-α and IL-1β levels increased by LPS. 3CPG and tricin did not affect NF-κB activation through phosphorylation and degradation of IκBα. However, 3CPG suppressed the production of NO and PGE_2_ by inhibiting iNOS and COX-2 expressions in LPS-stimulated RAW264.7 macrophages, respectively [29].

Regarding anti-obesity activity in 3T3-L1 cells, *p*-coumaric acid inhibited adipogenesis during the last stage of differentiation by downregulating the adipogenic transcription factors C/EBPa and SREBP-1c [32]. It activated the AMPK signaling pathway by enhancing the phosphorylation of AMPK and ACC, which can lead to fatty acid β-oxidation [32].

With respect to hepatoprotective activity, *p*-coumaric acid inhibited cellular lipid accumulation in HepG2 cells induced by oleic acid exposure by activating the AMPK pathway [33]. *p*-Coumaric acid significantly increased the gene expression of CPT-1a and phosphorylation of AMPK and ACC. Additionally, *p*-coumaric acid suppressed the expression of a transcription factor important in de novo lipid synthesis, SREBP-1c, and its target gene, FAS, which is involved in fatty acid biosynthesis [33].

Targeting of important pathways such as Wnt/β-catenin signaling, mitochondria-dependent apoptosis, NF-κB signaling, and AMPK pathways by SQLE and its phytochemicals supports the multiple pharmacological properties of SQLE. Additionally, it indicated the possible activities of SQLE against other diseases (such as neurodegenerative disorders) associated with these target pathways, which may be studied in future studies.

Importantly, some target genes/markers were found to be targeted by SQLE in different pharmacological activities (Figure 3). These common targets can be considered more important and reliable targets of SQLE activities; for example, activation of the AMPK signaling pathway through phosphorylation of the AMPK and ACC was observed in anti-obesity, anti-diabetic, and hepatoprotective activities of SQLE (Figure 3).

These targets are also targeted/affected by several drugs, which opened the possibility of SQLE interacting with other drugs. The possible drug interaction of SQLE may cause negative or positive outcomes. In a study, the synergistic effect of SQLE with the known cancer drug (cisplatin) was observed against cancer [24]. The synergistic effect was maybe due to the impact on the common target pathway, i.e., the PI3K/mTOR signaling pathway. Caution may be required when SQLE is used simultaneously with other drugs that impact the same targets as SQLE. Phytochemicals also interact with drugs by altering their metabolism and transport [70]. Cytochrome P450 is the major enzyme involved in the metabolism of drugs. Therefore, it is also suggested to study the impact of SQLE on CYP450 to explore possible drug interactions.

The pharmacological activity of *S. quelpaertensis* occurs during different developmental stages. SQLE showed effective activity against selected bacterial species and PRRSV. However, only a limited number of antibacterial studies have been reported; thus, more studies are required to establish the antibacterial activity of *S. quelpaertensis* against important bacterial pathogens.

Antiviral activity was analyzed only against PSSRV, which revealed the inhibition of inflammation, virus titer, viral protein, and viral RNA. The effective antiviral activities against PSSRV observed in diverse experiments emphasize the antiviral potential of SQLE. Therefore, the antiviral activity reported in future studies may be extended to other important pathogenic viruses, particularly RNA viruses. Notably, these antimicrobial activities were evaluated exclusively in vitro. Consequently, future research should utilize more physiologically relevant in vivo models to account for the bioavailability and pharmacokinetics of SQLE.

The tyrosinase inhibitory activity of *S. quelpaertensis* has been observed to produce an anti-melanogenesis effect, supporting its potential as a skin-whitening agent [15,36,37]. The formulations incorporating SQLEs are utilized across diverse commercial cosmetic product matrices, such as moisturizing creams, facial masks, topical serums, and hair care products. The utilization of SQLE by prominent Korean cosmetic manufacturers is primarily driven by its antioxidant, anti-tyrosinase, and skin whitening potential. However, these properties were studied only in vitro and in cell lines [15,36,37]. Further in vivo investigations are essential to validate the activity of SQLE and characterize its pharmacokinetic profile, both of which are critical for supporting its clinical translation.

A single in vitro study demonstrated the ADH- and ALDH-inducing activities of SQLE. While in vitro studies provide initial insights, their limitations—such as the lack of metabolic context—must be addressed through in vivo pharmacokinetic evaluations to ensure successful pharmacological development. Further studies, especially in vivo experiments, are required to establish ADH- and ALDH-inducing activity to develop *S. quelpaertensis* against alcohol-associated diseases and conditions [26].

The antioxidant activity of *S. quelpaertensis* may be considered its key activity that may contribute to its other important activities, including anticancer, hepatoprotective, and anti-UC activities. However, only a limited number of in vivo studies have analyzed the antioxidant potential of *S. quelpaertensis*. Furthermore, its antioxidant activity in different animal models suggests the antioxidant potential of *S. quelpaertensis*.

Anti-inflammatory activity is crucial for the protective effects of *S. quelpaertensis* in various organs, including the spleen [66], intestine [44,46,67], brain [8], liver [34,65], and heart [34,65]. However, further studies are necessary to establish the protective effects of *S. quelpaertensis* on organs such as the brain and heart. In different studies, its anti-inflammatory activities involved the inhibition of important cytokines and genes, which may protect other key organs such as the lungs and kidneys from inflammation-induced damage. Furthermore, the anti-inflammatory activity of SQLE may extend to other organs such as the bone, joints, pancreas, lungs, and kidneys.

The antioxidant and anti-inflammatory activities of SQLE have been shown to contribute to its effective anti-UC activity [44,46,67]. The role of the gut microbiome in the anti-UC activity of SQLE has been analyzed, revealing the restoration of gut microbial diversity and composition of gut microbiota [67]. However, in all studies, the anti-UC activity was evaluated in similar DS-induced anti-colitis models; thus, further studies using different models, such as genetically modified animals, and other UC-inducing chemicals, such as trinitrobenzene sulfonic acid. It may help evaluate the molecular mechanism of SQLE and establish its activity before clinical consideration.

Anticancer activity is considered a key activity of *S. quelpaertensis,* as it has been found to be effective against various cancers, including leukemia [22], lung [24], colon [25], and gastric cancer [4]. Furthermore, its antioxidant and anti-inflammatory activities have been shown to support the anticancer activity of SQLE. However, the major gap in the establishment of the anticancer effects of SQLE is the lack of in vivo studies for most cancers, such as leukemia, lung, and gastric cancer; this gap needs to be addressed to enable its further development as an anticancer agent. The anticancer activity of SQLE against most cancers should be evaluated in different animal models prior to clinical studies.

In one study, SQLE showed effective antidepressant activity in two behavioral tests, which was attributed to an increase in neurotransmitters (serotonin and dopamine) and the number of cells producing these neurotransmitters (TH-positive cells) in the brain. The suppression or dysregulation of neurotransmitters is associated with various brain diseases, including Alzheimer’s disease, Parkinson’s disease, and schizophrenia [71]. Further studies on the effect of SQLE against these neurological diseases are recommended. Notably, a high dose of SQLE was ineffective in this study, highlighting the need to identify the components of SQLE responsible for its activity. Identifying the components of SQLE responsible for its antidepressant activity could help optimize its antidepressant potential.

The anti-obesity activity of SQLE was observed in both in vitro and in vivo experiments. Reduction in lipid accumulation, adipose tissue weight, and body weight highlighted the anti-obesity potential of SQLE. Furthermore, AMPK signaling played a role in the anti-obesity effects of SQLE in in vivo and in vitro studies. The main constituent compounds of SQLE, such as tricin and *p*-coumaric acid, can target important pathways like AMPK. These specific compounds and SQLE are still mainly in the initial stages of drug development and have not yet reached widespread clinical application in humans. The anti-obesity effect achievable through the AMPK pathway, however, has been studied in human clinical trials. A clinical study of SQLE in Korean obese adults revealed marginal reductions in waist circumference, total abdominal fat mass, and triglyceride levels [72]. The optimization of active compound content, such as *p*-coumaric acid within the extract, targeting pathways like AMPK, and larger-scale clinical trials may yield significant reductions in obesity in future studies.

Hepatoprotective activity is one of the most studied activities of SQLE; it was found to be effective in both in vitro and in vivo experiments. SQLE and its important constituents suppressed intracellular lipid accumulation in HepG2 cells by activating the AMPK signaling pathway. Ethanol toxicity in HepG2 cells was suppressed through the reduction of ROS and enhancement of antioxidant enzymes such as catalase and GPX-1. Similarly, animal studies have emphasized the hepatoprotective activity of SQLE against ethanol toxicity through the healing of liver tissue injuries in immunohistology experiments, increased expression of antioxidants, and activation of the AMPK signaling pathway. These studies demonstrated the strong hepatoprotective potential of SQLE through effects such as the suppression of ROS, reduction in intracellular lipid accumulation, and an increase in antioxidant enzymes. These effects can protect the liver against different types of toxicants. However, in all animal studies, the hepatoprotective activity of SQLE was studied only against ethanol toxicity. Therefore, to further validate and explore the underlying molecular mechanisms, studies are recommended to evaluate the hepatoprotective activity against other models of hepatotoxicity using different toxicants, such as carbon tetrachloride (CCl4), thioacetamide, and acetaminophen.

Immune enhancement can contribute to the overall health of an organism, which includes fighting against infectious and non-infectious diseases. This is the main reason why herbal immune-enhancing supplements are in demand. In both in vitro and animal studies, *S. quelpaertensis* exhibited effective immunomodulatory activity. These studies evaluated a fermented product prepared from *S. quelpaertensis* and a combination therapy with *S. quelpaertensis*, which limited the exploration of the specific immune-enhancing activity of *S. quelpaertensis*. Thus, studies to evaluate the immunomodulatory activity of SQLE and its important constituents are strongly suggested to further develop *S. quelpaertensis* as an immune-enhancing supplement/therapeutic.

Notably, the pharmacological properties of culms have only been investigated in a few studies. Most studies have focused on analyzing the pharmacological properties of SQLE. However, *p*-coumaric acid, which is considered the key component responsible for the pharmacological properties of *S. quelpaertensis*, is more abundant in the culm extract than in the leaves [18]. The culm extract showed greater pharmacological effects, such as anti-melanogenesis activity, than the leaf extract [37]. Therefore, future studies should explore the pharmacological activity and the underlying mechanisms of the culm extract.

Multiple pharmacological activities of *S. quelpaertensis* can list *S. quelpaertensis* among popular medicinal plants such as green tea, ginseng, and *Curcuma longa* [73]. Potential biological activities such as immunomodulatory, anticancer, antioxidant, anti-inflammatory, anti-diabetic, antimicrobial, hepatoprotective, and anti-obesity activities are common in these plants and *S. quelpaertensis* [73]. In the cases of green tea, ginseng, and *Curcuma longa*, a high number of studies have established the medicinal potential of these plants. The comprehensive comparison of the biological properties of *S. quelpaertensis* with other important medicinal plants has not studied. However, a preliminary animal study has shown that the weight control was similar in the green tea and SQLE treatment groups, while the lowering of plasma triglyceride levels was better in the green tea group [2]. Comparative studies of critical biological properties like hepatoprotective and anticancer activities may be suggested in the near future.

General limitations in the current development of *S. quelpaertensis* across different pharmacological activities include the lack of robust pharmacokinetic studies, insufficient safety studies, and clinical trials. Pharmacokinetic studies of the extract are missing, which prevents a thorough exploration of the bioavailability of its active components. Furthermore, the use of high concentrations (up to 300 mg/kg) in some studies raises safety concerns due to potential toxicity or side effects. Conducting pharmacokinetic studies is therefore necessary to properly design dosage regimens and support subsequent in vivo and clinical development of SQLE.

Finally, it can be concluded that *S. quelpaertensis* exhibits potent pharmacological activity against various diseases and conditions. These activities are under different phases of investigation. The suggested directions can help the design of further in vivo and clinical studies on *S. quelpaertensis*.

## 7. Future Directions

While the various pharmacological activities of *S. quelpaertensis* are at differing stages of research development, each is characterized by specific knowledge gaps and challenges detailed in its respective section. This section outlines the critical challenges and proposes potential strategies to advance the pharmacological development of *S. quelpaertensis*.

- The absence of quantitative chemical profiling in many studies—where extracts remain poorly characterized—severely restricts the reproducibility and comparability of research. To address this, it is essential that future manuscripts provide detailed extraction protocols along with their standardization strategies, precise yields, and the quantification of specific marker compounds [8,9,22,24,25,34].
- While most research focuses on *S. quelpaertensis* leaves, the culm extract contains (or may contain) higher concentrations of the bioactive marker *p*-coumaric acid. Given its superior anti-melanogenic and pharmacological potency, future studies should prioritize investigating the pharmacological properties of culm.
- Pharmacokinetic and safety data for *S. quelpaertensis* are currently very limited; therefore, comprehensive studies in these areas are strongly warranted in the future.
- The scarcity of clinical data remains a critical bottleneck in the pharmacological development of *S. quelpaertensis*. Consequently, large-scale clinical trials are suggested to validate its therapeutic potential.

## Figures and Tables

**Table 2:** Pharmacological effects of *S. quelpaertensis* reported through in vitro studies.

Activity	Plant Part/Extract Type	Method	Result	Ref.
Anticancer activity	Methanolic SQLE	MTT of HL-60 cells	IC_50_ = 24.8 μg/mL. (ethyl acetate fraction)	[22]
		Nuclear condensation through fluorescence microscopy	Condense nuclei↑	
		Annexin-v and PI double staining	Apoptotic cells↑	
		WB	Expression of cleaved caspase3 and 9↑ and PARP degradation↑	
	Ethanol SQLE	MTT of HCT116 cells	49% inhibition at 200 μg/mL	[23]
	SQLE and its combination with CDP	MTT of A549 and H1299 lung cancer cells	A549↓ and H1299↓	[24]
		WB	Expression of MMP-2↓, phosphorylation of PI3K/AKT↑, and mTOR↑	
		CD44 and SOX-2 markers counts of CSC through flow cytometry	Population CSCs↓	
		Sphere formation assays	Size and number of the sphere↓	
		Clonogenic assays	The colony formation of CSCs↓	
		Wound Healing and Transwell invasion Assays	Wound healing and invasion assay migration of cells ↓	
		Gelatin zymography Assays	Enzymatic activity of MMP-2↓	
	SQLE and its major compounds (tricin and *p*-coumaric acid)	The CD133+ and CD44+ stained CSC isolated from the HT29 and HCT116 cell lines	Colony formation of CSCs↓ (with SQLE treatment at 200 and 300 μg/mL)	[13]
		Self-renewal assay	Number of spheres↓ (by the of SQLE, tricin and *p*-coumaric acid)	
		Clonogenic Assays	Colonogenic capacity↓	
		WB	DLK1↓, Sox-2↓, CD133↓, Notch1↓, HIF-1α↓, β-catenin↓, VEGF↓, CD44↓, and phosphoGSK3β↓	
		Real-Time Quantitative PCR	CD133, CD44, DLK1, Notch1, Sox-2, and VEGF	
	SQLE (100 μg/mL) and *p*-coumaric acid (2000 μg/mL)	MTT assay on MKN-74, MKN-45, HL-60, SNU-1, and SNU-16 cell lines.	MKN-74 cells↓ (81%) > SNU-16 cells↓ (80%) > HL-60 cells↓ (60%) > MKN-45 cells↓ (51%) > SNU-1 cells↓ (30%) by SQLE HL-60 cells↓ (88%) > SNU-16 cells↓ (48%) > SNU-1 cells↓ (40%) > MKN-74 cells↓ (25%) > MKN-45 cells↓ (21%) by *p*-coumaric acid	[4]
		Flow cytometry studies on HL-60 cells by measuring the amount of sub-G1 DNA.	Apoptosis induction through G1 arrest	
		WB of (HL-60 and MKN-74 cells)	Bax↑, Bcl-2↓, procapspase-3↓, and PARP↓	
	SQLE 25, 50, 100 and 200 μg/mL	Cell viabiliyt assays of HCT116 human colon carcinomas, wild-type p53 (p53-WT) and complete knockout of p53 (p53-null) cells	Cell viability of both type of cells↓	[25]
	SQLE 200 μg/mL	Apoptosis analysis through Annexin V apoptotic assay apoptosis of p53-WT and p53-null HCT116 cells	Apoptotic cells increased in time dependent manner. Induced apoptosis in both type of cells↑	
		Cell cycle analysis	Percentage of S and G2/M phases cells↑, while those in the G1 phase↓	
		Nitrite production measuring nitrite in media fractions by the Griess reaction	NO↑	
		Semi-quantitative RT-PCR analysis	Nitric oxide synthase NOS (eNOS↑, iNOS↑, and nNOS↑, CIAP-1↓, and CIAP-2↓	
		WB	Survivin↓, CIAP-1↓, CIAP-2↓, Bcl-2↓, and PARP↑	
		Caspase-3 activity assay in a colorimetric assay based on the cleavage of the synthetic peptide Ac-DEVD-pNA was performed	Caspase-3 activity↑	
Tyrosinase inhibitory activity	Compounds isolated from the Leaves extract	Tyrosinase inhibitory activity, estimated by measuring the amount of DOPA quinone	IC_50_ values were 0.027 and 0.026 mM for *N-p*-coumaroyl serotonin and *N*-feruloylserotonin, respectively.	[15]
	SQLE 80% ethanol. (125, 250, and 500 μg/mL)	Tyrosinase inhibitory activity, estimated by measuring the amount of DOPA quinone	>80% inhibition at 500 μg/mL	[16]
	SQLE	Tyrosinase inhibitory activity, estimated by measuring amount of DOPA quinone	51.00 ± 1.80%	[7]
Antimicrobial activity	Leaves extract of 0–100% ethanol extract.	MIC through the serial two-fold dilution method.	The MIC values (80% ethanol extract) were 500, 1000, 1000, 500, 250, and 1000 μg/mL for *Bacillus cereus*, *Staphylococcus aureus*, *Pseudomonas aerginosa*, *Escherichia coli*, *Proteus vulgaris*, and *Pichia jadnii*, respectively	[16]
	SQLE 1–5 mg/mL	PAM cell line (PAM-KNU) infected with PRRSV	Virus titer↓ (ED_50_ = 3.1 mg/mL)	[6]
		WB	PRRSV N protein	
		RT-PCR	Genomic and subgenomic mRNA↓, IL-1α↓, IL-6↓, IL-8↓, IL-15↓, TNF-α↓, AMCF-1↓, MCP-1↑, RANTES↑, interferon regulatory factors (IRFs), Toll-like receptors (TLRs)↑, Mx1↑, and ISG-15↑	
		NB	transcription↓	
ADH and ALDH enhancement activity.	Leaves extract of 80% ethanol and its fractions in ethyl acetate, chloroform, n-butanol, n-hexane, and water.	ADH activity assay	Ethyl acetate > chloroform > n-butanol > n-hexane > ethanol extract > aqueous (decreasing order of activity)	[26]
		ALDH activity assay	Ethyl acetate > chloroform > n-butanol > ethanol extract > aqueous > n-hexane (decreasing order of activity)	
Antioxidant activity	Methanol extract of leaves and its fraction in hexane, water, butnol, and ethyl acetate.	DPPH	IC_50_ values were 862.5 ± 6.4, 288.9 ± 12.7, and 166.4 ± 9.4 μg/mL for methanol, ethyl acetate, and butanol, respectively.	[27]
		Nitric oxide scavenging activity	IC_50_ = 259.4 ± 1.6 μg/mL (ethyl acetate fraction)	
		XOD-inhibition activity	IC_50_ values were 32.4 ± 1.6, 238.4 ± 5.6, 352.9 ± 16.0, and 473.5 ± 15.4 μg/mL for ethyl acetate, hexane, methanol, and butanol fraction, respectively.	
		Superoxide generation activity	IC_50_ values were 21.9 ± 5.4, 23.4 ± 5.9, 62.8 ± 4.3, 113.5 ± 13.4, and 305.2 ± 6.9 μg/mL for ethyl acetate, butanol, hexane, methanol, and water fraction, respectively.	[27]
	Leaves extract in ethanol and water	DPPH	EC_50_ values were 129.5 and 151.1 μg/mL for ethanol and water extracts, respectively.	[23]
		Hydrogen peroxide inhibition	EC_50_ values were 98.2 and 127 μg/mL for ethanol and water extracts, respectively.	
		Reducing power	EC_50_ values were 35.5 and 52.8 μg/mL for ethanol and water extracts, respectively.	
		Ferrous ion chelating	EC_50_ values were 81 and 31.7 μg/mL for ethanol and water extracts, respectively.	
		Nitric oxide scavenging	EC_50_ values were 114.7 and 142.7 μg/ for ethanol and water extracts, respectively.	
	Leaves extract in ethanol	DPPH	IC_50_ = 184.8 ± 2.9 μg/mL (December)	[18]
	Leaves extract in water		IC_50_ = 178.8 ± 10.8 μg/mL (November)	
	Culms extract in ethanol		IC_50_ = 785.5 ± 41.6 μg/mL (September)	
	Culms extract in water		IC_50_ = 748.5 ± 7.3 μg/mL (September)	
	Leaves extract in ethanol	ABTS	IC_50_ = 72.6 ± 3.6 μg/mL (December)	
	Leaves extract in water		IC_50_ = 88.8 ± 3.6 μg/mL (December)	
	Culms extract in ethanol		IC_50_ = 214.8 ± 15.7 μg/mL (August)	
	Culms extract in water		IC_50_ = 227.5 ± 79.8 μg/mL (September)	
	Leave extract in *n*-hexane, chloroform, *n*-butanol, and ethyl acetate fractions	DPPH	IC_50_ values were 42.9, 60.5, 152.6, 246.8, and 613.6 μg/mL in ethyl acetate, *n*-butanol, chloroform, ethanol extract, and *n*-hexane, respectively.	[17]
		Inhibitory activities of NO production in RAW 264.7 cells stimulated by LPS	Dose-dependent inhibition of NO production was observed	[17]
	Leaf extract with optimize conditions	DPPH	83.65 ± 1.56%	[7]
	Leaves extract in water at 50 μg/mL	Inhibition of ROS production in H_2_O_2_-stimulated PC12 neuronal cells	Decreased ROS production at 0.56 fold (in May)	[19]
Anti-inflammatory activity	The hot water extract of leaves	NO production in the LPS-induced RAW 264.7 cells	NO production↓	[28]
		Inducible isoform of nitric oxide synthase enzyme (iNOS)	iNOS activity↓	
		WB	iNOS↓	
		Luciferase based method the activation of NF-κB	NF-κB activation↓	
		Cell death in the RAW 264.7 cells observed through measurement of lactate dehydrogenase (LDH)	Cell death in RAW 264.7 cells decreased	
	3CPG from the leaves of SQLE	LPS induced RAW 264.7 cells NO production assay	NO production↓	[29]
		RTPCR	iNOS gene expression↓	
		ELISA	PGE_2_↓ and IL-6↓	
		WB	COX-2↓	
	SQLE	Co-culture medium consists of intestinal epithelial Caco-2 cells and RAW 264.7 macrophages	NO production↓	[30]
	100, 200, or 400 μg/mL),	ELISA	PGE_2_↓, IL-1β↓, and IL-6↓	
		WB	Hyperphosphorylation of IκBα↓, iNOS↓ and COX-2↓	
		RT-PCR	TNF-α↓	
	Leaves extract in ethanol	NO production in RAW 264.7 cells stimulated by LPS	IC_50_ = 39.07 ± 4.40 μg/mL (October)	[18]
	Leaf extract in water	NO production in RAW 264.7 cells stimulated by LPS	IC_50_ = 294.28 ± 10.92 μg/mL (August)	
	Culms extract in ethanol	NO production in RAW 264.7 cells stimulated by LPS	IC_50_ = 82.59 ± 17.89 μg/mL (October)	
	Culms extract in water	NO production in RAW 264.7 cells stimulated by LPS	IC_50_ = 353.54 ± 38.45 μg/mL (October)	
Anti-diabetic activity	SQLE	L6 rat skeletal muscle cell under hypoxic conditions and expression analysis through WB analysis.	Phosphorylated AMPK↓, phosphorylated acetyl-CoA carboxylase↓, and GLUT4↓	[31]
		L6 rat skeletal muscle cells glucose uptake through 2-NBDG uptake assay	Glucose uptake↑	
		Oil red-O staining and triglyceride content measurement	Triglyceride accumulation↓	
		WB	Phosphorylation of AMPK↑ Expression of CD36↑, fatty acid translocase (FAT↑), and transcription factor PPARγ↑, sterol regulatory element-binding protein (SREBP)-1c↓, FAS↓, acetyl-CoA carboxylase (ACC) phosphorylation↑	
Anti-obesity	SQLE	3T3-L1 cells WB RT-PCR 3T3-L1 cells	Phosphorylation of AMPK↑ and ACC↑ CPT-1a↑	[5]
	SQLE and its constituent *p*-coumaric acid	Oil Red O staining of 3T3-L1	Lipid accumulation↓	[32]
		WB	PPARc↓, C/EBPa↓, SREBP-1c↓, FAS↓ and aP2↓, and phosphorylation of AMPK↑ and ACC↑	
		RT-PCR	CPT-1a↑	
Hepatoprotective activities	SQLE	HepG2 cells induced by oleic acid and Oil red O staining	Lipid accumulation↓	[33]
		WB	Expression of SREBP-1c↓ and FAS↓ and phosphorylation of AMPK↑ and ACC↑	
		RT-PCR	CPT-1a mRNA↑	
	SQLE 60% ethanol extract	HepG2 cells toxicity induced by ethanol MTT assay	HepG2 cells viability↑	[34]
	SQLE fractions in n-hexane, chloroform, ethyl acetate, n-butanol, and distilled water.	HepG2 cells exposed to ethyl alcohol toxicity	HepG2 cells viability↑ (in ethyl acetate)	[20]
	Ethyl acetate fraction of SQLE	PI staining of HepG2 cells	Apoptotic DNA in the sub-G1 phase↓	
		Thymidine incorporation assay	Proliferation in HepG2 cells↑	
	SQLE fraction in n-ethyl acetate	MTT and colony-forming assays of HepG2 cells treated with ethanol	HepG2 cells viability↑	[9]
		PI staining of HepG2 cells	Apoptotic DNA in the sub-G1 phase↓	
		DCF-DA assay for ROS and Griess assay for NO	ROS↓ and NO levels↓	
		WB	Catalase↑ and GPX-1↑	
Immunomodulatory activity	Mixture of SQLE and FE in differeratiostio Mixture of SQLE and FE in different ratio	RAW264.7 cells macrophages	NO production↑	[35]
		ELISA	IL-1β↑, IL-6↑, and TNF-α↑	

2-NBDG: 2-[N-(7-nitrobenz-2-oxa-1,3-diazol-4-yl) amino]-2-deoxyglucose; ABTS: 2,2′-azino-bis(3-ethylbenzothiazoline-6-sulfonic acid; 3CPG: 3-*O*-*p*-Coumaroyl-1-(4-hydroxy-3,5-dimethoxyphenyl)-1-*O*-β-D-gulcopyranosylpropanol; ADH: alcohol dehydrogenase; ALDH: aldehydedehydrogenase; AKT: protein kinase B; CDP: Cis-diammineplatinum II; DPPH: 2,2-diphenyl-1-picrylhydrazyl; DOPA: l-3,4-dihydroxyphenylalanine; ELISA: enzyme-linked immunosorbent assay; EC_50_: half maximal effective concentration; FE: *Ficus erecta*; IC_50_: half maximal inhibitory concentration; LPS: lipopolysaccharide; PGE_2_: inflammatory prostaglandin E_2_; PI3K: phospholrylation of phosphoinosi-tide-3 kinase; IL: interluekin; mTOR: mammalian target of rapamycin; iNOS: inducible isoform of nitric oxide synthase enzyme; LDH: lactate dehydrogenase; MIC: minimun inhibitory concentration; MTT: 3-(4,5-di methyl thiazol-2-yl)-2,5-diphenyltetrazolium bromide; NF-κB: nuclear Factor kappa B; NO: nitric oside; ROS: reactive oxygen species; RT-PCR: real time polymerase chain reaction; DCF-DA: 2,7-dichlorofluorescein diacetate; PI: propidium iodide; SQLE: *Sasa quelpaertensis* leaf extract; CSC: Counts of cancer stem cells; p53-WT: wild-type p53; p53-null: complete knockout of p53 cells; WB: Western blot; XOD: xanthine oxidase; ↑: up-regulation; ↓: down-regulation.

**Table 3:** Pharmacological effects of *S. quelpaertensis* reported through in vivo studies.

Activity	Plant Part/Extract Type	Method	Result	Ref.
Anticancer activity	SQLE 300 mg/kg BW orally 5 times a week for three weeks	Male BALB/c nude mice xenograft model through subcutaneous injection of CD133+ and CD44+ double-stained HT29 cells.	Mean volume of tumor↓ *	[13]
		WB	DLK1↓, Sox-2↓, CD133↓, Notch1↓, HIF-1α↓, β-catenin↓, VEGF↓, CD44↓, and phosphoGSK3β↓	[13]
Antidepressent	Leave extract (100 and 300 mg/kg) for 14 consecutive days.	Ovariectomized Sprague Dawley (SD) rats in the tail suspension test and forced swim test.	In both TST and FST tests, the immobility time↓	[8]
		ELISA analysis for serotonin and dopamine in different parts of the brain	Dopamine↑ (in all brain), serotonin↑ (in the prefrontal cortex (PFC) and hypothalamus regions)	
		Immunohistochemistry analysis for TPH, TH, and PKC.	TH positive cell↑, hypothalamic TPH+ cells↑, TPH-IR cells↑ (in the Peduncular part of the lateral hypothalamus), and PKC-IR cells↑ (in the hypothalamic Area)	
Anti-fatigue	SQLE 50 mg/kg body weight + exercise for 7 days	Male ICR mice exhaustive swimming test	Average of swimming time↑	[59]
		Blood glucose and lactate, and muscular lactate and glycogen levels were studied	Levels of blood glucose↑, blood lactate↓, muscular lactate↓, and muscular glycogen↑	
		Comparative RNA-Seq of the muscular transcriptome and enrichment analysis	835 genes were found to be differentially expressed and enriched in biological processes, such as glycogen metabolism, lipid metabolism, carbohydrate metabolism, reactive oxygen species metabolism, and transport. In pathways, AMPK signaling, insulin signaling, carbon metabolism, endocytosis, and cytokine–cytokine receptor interaction were the enriched pathways.	
Anti-ulcerative colitis	SQLE (100 and 300 mg/kg) of SQLE	Male C57BL/6 mice	DAI score↓, and colon length↑	[44]
		Histopathology	Tissue damage↓	
			PCNA-positive cells↓	
		RTP-CR	TNF-α↓	
		WB	TNF-α↓, cytosolic iNOS↓, and COX-2↓ phosphorylation of IκB↓, ERK↓, JNK↓, and p38↓	
	SQLE (100 and 300 mg/kg) of SQLE	Male C57BL/6 mice	DAI score↓, and colon length↑	[46]
		Histopathology	Tissue damage↓ and crypt distortion↓	
		Gut motility	Whole gut transit time↓	
		The DNA damage in the colon tissues8-oxo-dG positive cell	DNA damage↓	
		The MDA level of plasma	MDA↓	
		The plasma SOD activity and catalase activity of the colon	The plasma SOD↑ activity and catalase activity↑ of the colon	
		WB	SOD1↑, SOD2↓, and GPx↓	
	SQLE 300 mg/kg of SQLE	Male C57BL/6 mice	DAI score↓, and colon length↑	[67]
		Gut microbiome analysis	OTUs↑, Chao1 estimator↑, and Shannon diversity index↑, ratio of Bacteroidetes to Firmicutes↓	
Anti-obesity	150 mg/kg of SQLE per day for 70 days	High-fat diet induced obesity in C57BL/6 mice.	BW↓, weights of epididymal↓ and perirenal adipose tissue↓	[5]
		Histological and H&E staining	Size of epididymal adipocytes↓ and accumulation of lipid droplets↓	
		Lipid profiling	Levels of TC↓, TG↓, GOT↓, LDH↓, and GPT↓ in the serum	
		WB	Phosphorylation of AMPK↑ and ACC↑ (in the liver tissues and epididymal adipose tissues)	
		RT-PCR	Adiponectin (in epididymal adipose)↑	
	SQLE supplementation in two doses (5% and 3%) for 11 weeks	Male Sprague Dawley (SD) rats’ plasma lipid profiling.	TC↓, TG↓, LDL-C↓, and AI↓ values were significantly suppressed, and HDL-C.↑	[62]
		ELISA	Plasma resistin level↓, plasma adiponectin levels↑ *	
		Lipid profiling liver	TG↓ and total lipid↓ levels	
		Histology analysis	Size of fat droplets↓, infiltration of inflammatory cells↓, and swelling of hepatic cells↓	
		WB	FAS↓	
		RT-PCR	Liver SREBP-1↓, liver UCP-2↓, heart resistin level↓, in visceral adipose tissue PPAR↓ *, C/EBPb↓, aP2↓ *, and SREBP-1↓ *	
Hepatoprotective activities	Female C57BL/6 mice SQLE (100 mg/kg BW)	Three intraperitoneal doses of 30% ethanol at 12 h intervals.	AST↓ and ALT↓	[65]
		TBARS assay	Lipid peroxidation↓ (in liver tissue)	
		Histology study of liver tissues	Increased content of lipid vacuoles↓, loss of membrane integrity↓	
	Butanol fraction of SQLE 30 mg/kg	Liver damage markers	AST↓ and ALT↓	
		TBARS assay	Lipid peroxidation↓ (in liver tissue)	
		Glutathione colorimetric assay of liver	GSH↑	
	80% ethanol SQLE (10 mg and 100 mg/kg BW	hepatotoxicity caused by the ethanol on the binge model of alcohol consumption.	The alcohol content of serum↓	[9]
		hematoxylin-and-eosin (H&E) staining	Steatosis of liver↓, increase in the lipid droplets↓, ballooning degeneration↓, and loss of cellular boundaries↓.	
		TBARS assay	lipid peroxidation in liver tissue↓	
		Glutathione colorimetric assay	The expression of the antioxidant GSH in the liver↑	
		Immunohistochemical analysis	CYP2E1↓	
		WB	CYP2E1-	
	10, 50, and 100 mg/kg BW	H&E staining of hepatotoxicity caused by ethanol in the binge model of alcohol consumption	Steatosis of liver↓, increase in the lipid droplets↓, ballooning degeneration↓, and loss of cellular boundaries↓	[20]
		TBARS assay	Lipid peroxidation in liver tissue↓	
		Glutathione colorimetric assay	GSH↑ (in the liver)	
		Immunohistochemical analysis	CYP2E1↓ and perilipin-2↓	
		WB	CYP2E1↓, TNF-α↓, AMPK↑, and FAS↓	
Immunomodulatory activity	Male BALB/c mice. Three doses (50, 100, and 200 mg/kg) of the combination of SQLE and FE (2:8 ratio)	The cytotoxicity activity of natural killer cells was studied in splenocytes that were co-cultured with YAC-1 cells (target cells)	Cytotoxicity activity of NK cells↑	[35]
		ELISA spleen	IL-2↑, IL-4↑, IL-10↑, IL-12↑, and TNF-α↑	
		Peritoneal exudate cell (PEC),	NO↑	
		FACS analysis for spleen, Peyer’s patches, draining lymph node, thymus, and PEC was studied	Number of total cells↑ (in spleen, Peyer’s patches, draining lymph node, and PEC), CD3+ T cells↑ (in spleen, DLN, and Peyer’s patch), CD4+ cells↑ (in thymus), CD49b+ NK cells↑ (in spleen, DLN), CD4+ Th cells↑ (in spleen, DLN), CD8+ T cells↑ (in spleen, DLN), CD4+/CD25+ Treg cells↑ (in spleen, DLN), CD23+/B220+ B cells↑ (in spleen), DLN Gr-1+/CD11b+↑ (in spleen), CD107a↑ (in spleen), CD3+/CD4+ Th cells↑ (in PEC and Peyer’s patch), CD3+/CD8+ Tc lymphocytes↑ (in PEC and Peyer’s patch), CD8+/CD25+ Treg cells↑ (in PEC), B220+/CD69+ B cells↑ (in PEC), B220+/CD23+ B cells↑ (in PEC), and CD11b+/CD69+ cell↑ (in PEC)	
	Balb/c male mice	Fermented product of SQL for 2 weeks cell population in PBMCs	Cell Population in PBMCs after the treatment showed a significant increase in the population of Helper T cells, Th cells, and cell-surface protein expression of CD4+CD8− (Helper T cells, Th cells) in three types of fermentation conditions.	[68]
		Macrophage population↑ in the peritoneal cavity	Macrophage population↑ (in the peritoneal cavity)	
		NO production macrophage population in the peritoneal cavity	NO production↑	
		The production of cytokines in splenocytes through ELISA	IL-4↑, IL-10↑, and IFN-γ↑	
Bone protective	OVXR female Sprague Dawley rats 10% of SQLP for 4 weeks	Femur BMC, BMC, and bone width were analyzed	BMC↑, and BMD↑	[66]

*: not significant; AI: atherogenic index; BMC: bone mineral concentration; BMD: bone mineral density; BW: body weight; DAI: disease activity index; GOT: glutamic oxaloacetic acid transaminase; GPT: glutamic-pyruvic acid transaminase; H&E: hematoxylin-and-eosin; HDL-C: high-density lipoprotein cholesterol; LDH: lactic dehydrogenase; LDL-C: low-density lipoprotein cholesterol; MDA: malondialdehyde; RNA-Seq: RNA sequencing; TH: tyrosine hydroxylase; TPH: tryptophan hydroxylase; PBMC: peripheral blood mononuclear cells; PCNA: proliferating cell nuclear antigen; PKC: Protein Kinase C; PFC: prefrontal cortex; OTUs: operational taxonomic units; OVXR: ovariectamized rats; TBARS: thiobarbituric acid reactive substances; TC: total cholesterol; TG: triglycerides; ↑: up-regulation; ↓: down-regulation.

## Data Availability

No new data were created or analyzed in this study. Data sharing is not applicable to this article.

## Abbreviations

The following abbreviations are used in this manuscript:

SQCEE	*Sasa quelpaertensis* culm ethanol extract
SQLEE	*Sasa quelpaertensis* leaf ethanol extract
SQLE	*Sasa quelpaertensis* leaf extract
SQLEEEA	Ethyl acetate fraction of *Sasa quelpaertensis* leaf ethanol extract
SQLEMEA	Ethyl acetate fraction of *Sasa quelpaertensis* leaf methanol extract
SQLP	*Sasa quelpaertensis* leaf powder
SQLFP	Fermented product of *Sasa quelpaertensis* leaves
ADH	Alcohol dehydrogenase
ALDH	Aldehyde dehydrogenase
BW	Body weight
DAI	Disease activity index
OVXR	Ovariectomized rats
TBARS	Thio-barbituric acid reactive substances
NO	Nitric oxide
ROS	Reactive oxygen species
RT-PCR	Real-time polymerase chain reaction
CSC	Counts of cancer stem cells
p53-WT	Wild-type p53
p53-null	Complete knockout of p53 cells
WB	Western blot
XOD	Xanthine oxidase
